# Immune cell counts and signaling in body fluids of cows vaccinated against *Clostridium difficile*

**DOI:** 10.1186/s40709-018-0092-4

**Published:** 2018-12-10

**Authors:** Christiane Schmautz, Nadine Müller, Marlene Auer, Ines Ballweg, Michael W. Pfaffl, Heike Kliem

**Affiliations:** Chair of Animal Physiology and Immunology, Technical University of Munich (TUM), Weihenstephaner Berg 3, 85354 Freising, Germany

**Keywords:** Gene expression, Cytokines, Dairy cattle, Immunization, *Clostridium difficile*

## Abstract

**Background:**

New treatment options are needed to prevent relapses following failed antibiotic therapies of *Clostridium difficile* infections (CDI) in humans. The concomitant therapy with an anti-*C. difficile* IgA containing whey protein concentrate can support the sustainable recovery of CDI patients. For 31 weeks, nine dairy cows were continuously vaccinated with several anti-*C. difficile* vaccines by certain routes of administration to produce anti-*C. difficile* IgA enriched milk. The study aimed at finding decisive differences between low responder (LR) and high responder (HR) cows (> 8.0 µg ml^−1^ total milk *C. difficile* specific IgA) concerning their immune response to vaccination on cellular and molecular biological levels.

**Results:**

The results of total and differential cell counting (DCC) in blood and milk and the outcomes of the gene expression analysis of selected immune factors were assessed relating to the usage of two vaccine batches for injection (*MucoCD*-*I* batch A and B), marking two immunization (IM) periods, and compared to a control group (Ctr). The *MucoCD*-*I* batch A caused short-term leukopenia followed by leukocytosis in the blood of LR and HR. The total somatic cell counts in milk were not altered by the treatment. The DCC revealed that the leukocytes of the treated groups were partly impaired by the treatment. The gene expression analysis exposed cumulative and sustainable differences (*p* < 0.05) between LR and HR for the genes encoding for *lactoferrin*, *CXCL8*, *IL1β*, *IL2*, *IL6*, *IL12β*, *IFNγ*, *CD4* and *CD163*. The regulation of the epithelial IgA cell receptor *PIGR* was not impaired by the IM. In contrast to the vaccination with *MucoCD*-*I* batch A, the second IM period with *MucoCD*-*I* batch B resulted in mitigation and synchronization of the treated groups’ immune responses.

**Conclusions:**

The inversely regulated cytokines in the blood and milk cells of the treated groups led to a variously directed, local T cell response resulting in their different production intensities of *C. difficile* specific IgA in milk.

**Electronic supplementary material:**

The online version of this article (10.1186/s40709-018-0092-4) contains supplementary material, which is available to authorized users.

## Background

*Clostridium difficile* is a gram-positive, endospore-forming and fastidious anaerobe bacterium [1]. The majority of *C. difficile* isolates is toxigenic and produces two large exotoxins, known as *C. difficile* toxin A (TcdA) and toxin B (TcdB), with enterotoxic and cytotoxic activities. In some epidemic strains (such as the ribotypes 078 and 027) a third toxin, the binary toxin *C. difficile* transferase, is related to germ virulence [2, 3]. *Clostridium difficile* endospores are widely disseminated in the environment, although the primary habitat of the vegetative form is the gastrointestinal tract of humans and animals [4]. An interference of the microbiome in the gut, for instance due to the intake of antibiotics, an impaired mucosal barrier, as symptomatic of inflammatory bowel disease (IBD), and, generally, a weakened immune system (IS) are the prominent risk factors of susceptibility to *C. difficile* infection (CDI) [2, 5]. In 2015, the U.S. Centers for Disease Control and Prevention (CDC) classified *C. difficile* as an urgent antibiotic resistance threat [6]. CDI is deemed to be the leading cause of health care-associated diarrhea, but according to the latest whole genome sequencing studies, CDI is mainly community-acquired and may have a foodborne or zoonotic etiology [1, 7, 8]. Serious and fatal progressions of this disease have been increasing for the last two decades [9]. The risk to relapse or to re-infect after the first finalized episode of CDI is in the range of 20 to 25% and continues to grow with every further episode [10, 11]. The best practice advices for managing the CDI, particularly in IBD, comprise treatment with vancomycin or fidaxomicin instead of metronidazole. To recover the colonization resistance, fecal microbiota transplantation should be recommended to patients with recurrent CDI [12, 13]. Another way to reduce recurrences is additional passive immunization of CDI patients. Anti-*C. difficile* specific antibodies directed against *C. difficile* main virulence factors, TcdA and TcdB, were concomitantly applied to antibiotic therapy and proven effective to prevent renewed *C. difficile* associated disease [1, 14, 15]. Therefore, a whey protein concentrate enriched with anti-*C. difficile* specific antibodies is an appropriate dosage form [16–18]. The targeted concentration of anti-*C. difficile* specific antibodies in bovine milk requires a continuous vaccination of dairy cows against the characteristic features of the microbe. Following vaccination, a complex set of interacting factors governs the development of systemic and secretory immune responses in vivo [19]. Exogenous factors include the characteristics of the vaccine, the route of administration and the possibly utilized immunologic vehicles for vaccine delivery such as adjuvants. Furthermore, environmental factors, psychological stress, nutrition and infectious diseases may have an impact on the vaccination response, whereas genetic factors, age and sex determine the intrinsic capacity of the recipients to respond to a vaccine [20]. Hence, the outcome of their immune reaction to the same immunological stimulus may vary fundamentally. Given this fact, high responder (HR) and low responder (LR) cows were identified, as measured by their production of anti-*C. difficile* specific antibodies in milk [21]. In the study at hand, the source of different immunological reactivity of the treated cows was examined within selected endogenous factors, as the exogenous factors influencing an active immunization were standardized by the experimental setup. In any case, vaccines are antigens and the in vivo challenge with an antigen causes a cellular reaction and leads ideally to a humoral response of the IS. The first encounter with the antigen provokes the innate IS, which includes components like the complement system or antigen-presenting cells (APCs). The latter activate the second arm of the IS, the adaptive IS, which is associated with lymphocytes as effector cells, antibody production and immunological memory [22]. The repeated exposure to the same antigen heightens these lymphocyte functions [23]. In the current study, the activation of the cellular stimulation by vaccination was assessed in blood and milk by the use of total and differential cell counting (DCC) as diagnostic tool for progress monitoring [24, 25]. A more fine-tuned cause analysis for the determined different responsiveness of the treated cows was exemplarily achieved by molecular genetic analysis to verify the degree of cellular activity by the expression of genes coding for specific surface determinants and for typical chemokines [26–30].

## Results

### *Clostridium difficile* specific IgA in milk

The full description of the *C. difficile* specific IgA contents in milk and their development over the first 31 treatment weeks (TWs) was disclosed by Schmautz et al. [21]. In summary, all cows designated for immunization (IM) showed a basic level of less than 2 µg of anti-*C. difficile* IgA per milliliter milk on average owing to the natural dissemination of *C. difficile*. The repeated IM challenged the IS of the treated cows, thereby triggering the production and release of specific antibodies in milk, *inter alia*. Since the extent of anti-*C. difficile* IgA enrichment in milk differed significantly between the immunized cows, they were classified into “low responder” (LR) and “high responder” (HR) by a threshold of 8.0 µg ml^−1^ *C. difficile* specific IgA in milk, as published by Schmautz et al. [21]. On completion of their above-mentioned 31-week treatment period, LR cows produced ordinarily 3.1 ± 0.3 µg and HR cows 9.6 ± 0.3 µg of anti-*C. difficile* IgA per milliliter milk, respectively. The initial applications of the two tested vaccines for injection, *MucoCD*-*I* batches A and B, HR accomplished peak values of roughly 15 µg anti-*C.* *difficile* IgA per milliliter milk, whereas LR never exceeded 5 µg ml^−1^.

### Gene expression data

In order to assess possible gene regulatory effects by IM in PBL and SCC, the gene expressions of different cell type marking receptors as well as immunologically relevant factors like chemokines were analyzed. A more detailed description of all analyzed genes is shown in Additional file 1: Table S1. The relative changes in gene expression were determined in relation to the baselines before the first-time application of the *MucoCD*-*I* vaccine batches [TW 0 (for PBL and SCC) and TW 15 (only for SCC) or TW 16 (only for PBL)]. The delta values, representing the gene expression changes measured in PBL and SCC, are accessible in Additional file 2: Table S2 and Additional file 3: Table S3, respectively. Table 1 shows the grading system being applied for the gene expression data.

**Table 1:** Classification of the gene regulation expressed as ΔΔCq range

Defined grades of gene regulation		ΔΔCq range
0	Non-regulated	| < 1|
+	Weak	| ≥ 1| up to | < 3|
++	Strong	| ≥ 3| up to | < 5|
+++	Very strong	| ≥ 5|

The grades of gene regulation are given as follows: “0” for non-regulated, “+” for weakly regulated, “++” for strongly regulated, “+++” for very strongly regulated

In favor of a structured compilation, the determined gene regulations were classified depending on their scale of the ΔΔCq range during the 31 TWs. The TW, marking the maximal significant up- or down-regulation of the investigated gene expression in relation to the belonging baseline, decided about the affiliation to one of the four grades of gene regulation (0, +, ++, +++). The group (HR, LR, control), in which the maximal change of gene expression was determined, did not matter for the classification. Table 2 summarizes the analyzed genes in PBL and SCC plus the information described above according to their cell type related affiliation.

**Table 2:** Spectrum of genes analyzed in PBL and in SCC out of milk and the classification of their regulation

Genes	Performed gene expression analysis		Classification of the gene regulation	
	In PBL	In SCC	In PBL	In SCC
bMEC′-related genes				
KRT8	No	Yes	/	+
PIGR^a^	No	Yes	/	++
FcRN^a^	No	Yes	/	0
C1QA^a^	No	Yes	/	0
C3^a^	No	Yes	/	+
CCL5^a^	No	Yes	/	+
CCL20^a^	No	yes	/	++
CCL28^a^	No	Yes	/	+
CXCL3^a^	No	Yes	/	+
CXCL5^a^	No	Yes	/	0
CXCL8^a^	No	Yes	/	+
LPO^a^	No	Yes	/	++
LF^a^	No	Yes	/	+
LYZ1^a^	No	Yes	/	+
TAP^a^	No	Yes	/	+
Phagocyte-related genes				
C3AR1	Yes	Yes	+	+
C5AR1	Yes	Yes	+	++
CXCR2	Yes	Yes	+	+
TLR2	Yes	Yes	+	+
CD163	Yes	Yes	+	0
CXCL8	Yes	See above	+	See above
TNFα	Yes	Yes	+	++
IL1β	Yes	Yes	++	+
IL6	Yes	Yes	+++	+
IL12β	Yes	Yes	++	+
Lymphocyte-related genes				
CD3δ	Yes	Yes	+++	+
CD4	Yes	Yes	++	++
CD8β	Yes	Yes	++	+
CD19	Yes	Yes	+	+
CD38	No	Yes	/	+
CD126	Yes	Yes	+++	+
CCR6	No	Yes	/	+
IFNγ	Yes	Yes	+	+
TGFβ1	Yes	No	+	/
IL2	Yes	No	+++	/
IL10	Yes	Yes	+	++

*PBL* peripheral blood leukocytes, *SCC* somatic cell count, *bMEC* bovine epithelial cells

^a^ characterizes the bMEC-related genes, which were additionally normalized with KRT8 as the epithelial cell marker; the sign / means that the gene expression was not analyzed in the study objects, PBL or SCC

### Phagocyte-related genes in PBL

The investigated receptors of phagocytes (*C3AR1*, *C5AR1*, *CXCR2*, *TLR2* and *CD163*) were regulated on a weak level in PBL. During the first IM period up to TW 14, only the complement receptor *C3AR1* in PBL of the LR was found up-regulated within the group and to the control group (Ctr) in TW 8. Following treatment with vaccine batch B, the HR showed a steadily increased *C3AR1* expression in PBL with significant differences to the Ctr on four dates, and additionally, to the LR in TW 23. *C5AR1*, the counterpart of the complement fragment C5a, was down-regulated in PBL of all treated cows directly after the injection of vaccine batch A. After the LR group *C5AR1* expression turned upward, it exceeded that of the HR group by achieving the ΔΔCq value of 1.7 in TW 10. Furthermore, the *C5AR1* expression differed between the treated groups in TW 14. At the second IM period, the expressions of *C5AR1* developed similarly toward its down-regulation by all treated cows from TW 21, and therefore the *C5AR1* transcript amount of HR was significantly lower than that of the Ctr in TW 23. The expression of the PMN specific chemokine receptor *CXCR2* was lastingly diminished in the treated groups after the application of *MucoCD*-*I* vaccine batch A. The treatment with vaccine batch B led to the enhanced transcription of *CXCR2* in HR compared to the LR in TW 18 and to the Ctr group in TW 20. In association with the vaccine batch A, the toll-like receptor 2 (*TLR2*) was down-regulated by half in the PBL of the treated groups in TW 4. Comparing HR and LR, *TLR2* was significantly down-regulated in the HR group in TW 8. During the second IM period, both treated groups exhibited an identical up-regulation of the *TLR2* transcription compared to the Ctr. The monocytes/macrophages specific surface determinant *CD163* was found significantly up-regulated in the LR compared to the HR (TW 2). Similar declines of *CD163* transcripts were determined in PBL of HR and LR in TW 4, the result of the previously given vaccine batch A on the PC route. Afterwards, the *CD163* expression by the HR PBL was mainly reduced up to TW 14 and it differed markedly from that in the LR PBL in TWs 8, 10 and 14. The second treatment period did not cause any alterations of the *CD163* expressions. The genes coding for the phagocyte-related cytokines, *CXCL8* and *TNFα*, were graded as weakly regulated in PBL. Following vaccination with the *MucoCD*-*I* batch A, *CXCL8* was more up-regulated in the PBL of HR than of LR, and its expression varied significantly between both treated groups at the TWs 5–7, 10, 12 and 14. The increased transcription of *CXCL8* by the Ctr, contrary to the lower one by the LR, led to clear differences between both groups in the TWs 10 and 12. As from TW 16, the second IM period was marked by reduced amounts of *CXCL8* transcripts, up to one-fifth compared to the baseline value in PBL of the Ctr group. Consequently, considering the scarcely altered *CXCL8* expression levels of the treated groups in the same time period, many times of investigation had significant discrepancies between them and the Ctr group. The amounts of *TNFα* transcripts were not impaired in any group during the first vaccination period, whereas *TNFα* was slightly up-regulated within all groups at various TWs during the use of vaccine batch B. Different *TNFα* expressions between HR and LR were not measured, but those of the treated groups were significantly up-regulated toward the Ctr group *TNFα* expression in TW 27. The investigated interleukins, *IL1β* and *IL12 β,* were proven as strongly regulated, and especially, the expression of *IL6* was very strongly affected by the IM. Within the HR group, a down-regulation of *IL1β* in PBL proceeded during the first 2 months of treatment. In relation to the baseline, the lowest point of *IL1β* expression was reached with remaining six percent of *IL1β* transcripts in TW 8. In contrast to the HR, the LR group’s *IL1β* expression was depressed to a lesser extent in PBL during the same time period. Therefore, differently pronounced down-regulations of *IL1β* were proven between them in TWs 1, 3 and 8. Comparing the *IL1β* gene regulations between the treated cows and the Ctr cows, considerable differences were revealed at numerous measurement points up to TW 14. In the ensuing second treatment period, the *IL1β* transcriptions of HR and LR were down-regulated only once in TW 20 and TW 21, respectively. On these dates, the measurement data of both treated groups deviated from each other and from the Ctr group, too. The transcription of *IL6* varied between the HR and LR in PBL in TWs 8 and 10 due to a partly weak *IL6* down-regulation in HR PBL. At the end of this IM period, the relative quantity of *IL6* transcripts of both treated groups undercut that of the Ctr group. In contrast to the first IM period, treatment with vaccine batch B evoked at least strong up-regulations of *IL6* in all treated cows, which were very strongly pronounced in the LR in TW 27. To this date and subsequent to the first PC injection of vaccine batch B, the *IL6* expression by the LR PBL surpassed clearly that of the HR. The courses of *IL12β* expressions were generally similar to the treated groups during both IM periods. Nevertheless, the HR and LR expressed *IL12β* in PBL at distinguishable levels during the first IM period, where the relative amounts of *IL12β* transcripts in LR PBL were larger than that in HR PBL at the TWs 1–3, 8 and 10. The LR showed a weak up-regulation of *IL12β* up to the application of *MucoCD*-*I* batch A, which was followed by a slight down-regulation only in TW 4, and a strong up-regulation of *IL12β* developed after TW 7. From TW 21, the expressions of *IL12β* in PBL of all treated cows were reduced to more than a quarter in the remaining second IM period.

### Lymphocyte-related genes in PBL

**Fig. 1:**
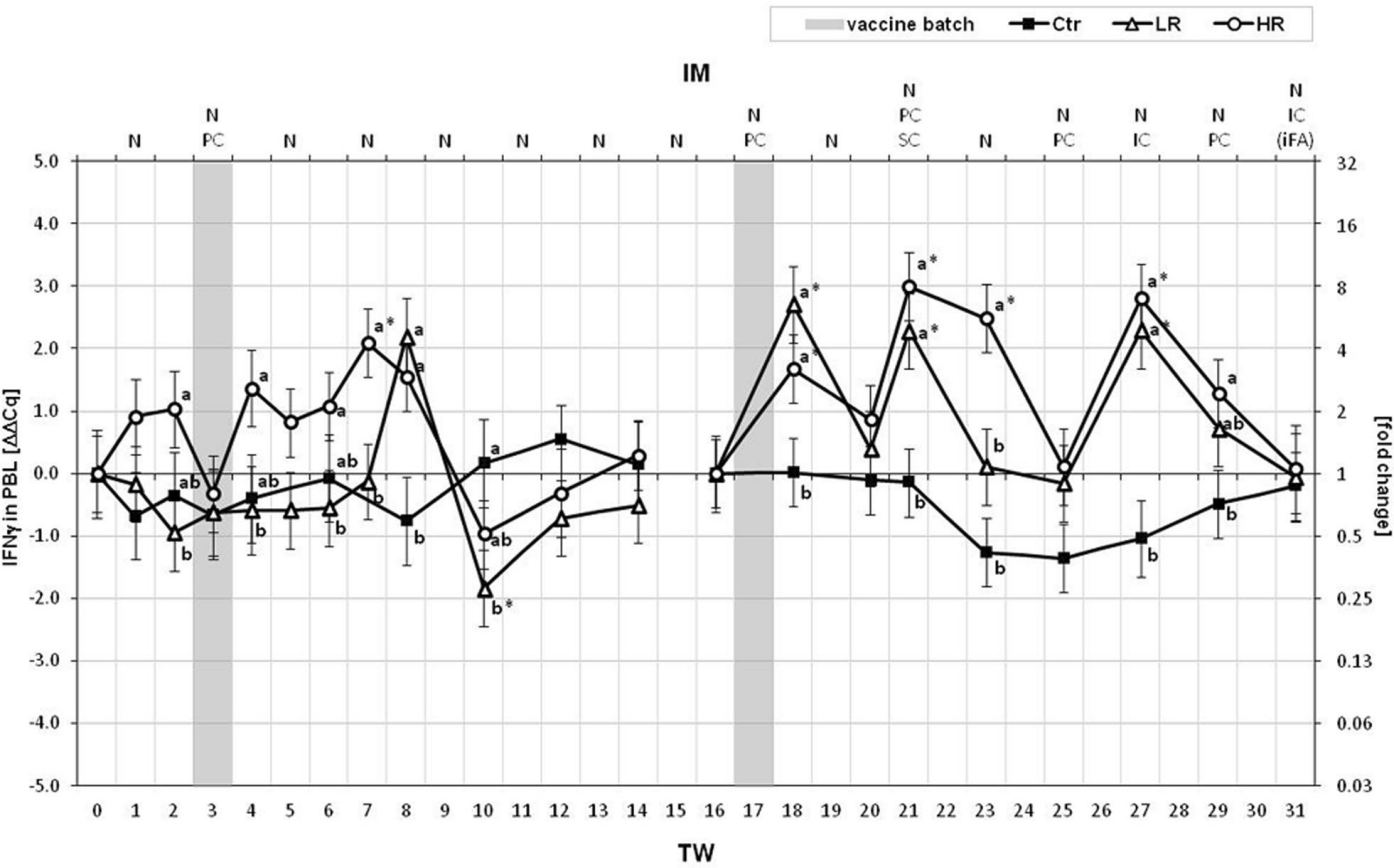
IFNγ gene expression in PBL. The ΔΔCq values of IFNγ as LSM ± SD and the corresponding fold changes are mapped on the ordinates for the control (“Ctr”, n = 5) and the treated animal groups (“LR”, low responder cows, n = 4; “HR”, high responder cows n = 5). The lower X-axis shows the 31-week treatment period (TW). The upper X-axis depicts the immunization (IM) specified by the routes of vaccine application [nasal (N), percutaneous (PC), subcutaneous (SC) and intracutaneous (IC)] per TW. Incomplete Freund’s adjuvant (iFA) was solely part of the *MucoCD*-*I* vaccine in TW 31. The gray bars in TWs 3 and 17 highlight the times of the first uses of vaccine batches A and B, respectively. During the treatment periods with both vaccine batches and the particular TW before the first use of vaccine batch A or B, substantial differences within the same group are marked by asterisks (*p* < 0.05). Lower case (a, b, c) indicate evident differences (*p* < 0.05) between the groups on the same dates

By focusing on the acquired IS, *CD3δ*, a part of the T cell antigen receptor (TCR), was mainly uniformly regulated in HR and LR. During the first IM period, *CD3δ* was slightly more expressed in PBL of the LR group compared to the other groups in TW 10 only. From TW 20, *CD3δ* was found very strongly down-regulated in HR and LR PBL compared to Ctr in relation with the combined PC/SC injection of *MucoCD*-*I* batch B. The expression of *CD4*, the T-helper (T_H_) cell-specific co-receptor for major histocompatibility complex (MHC) class II molecules, was graded as strongly regulated in the PBL of the treated groups. From TW 7, four TWs after the vaccination with *MucoCD*-*I* batch A, the *CD4* expression was lastingly depressed in PBL of the HR. Consequently, the HR relative level of *CD4* transcripts was distinct towards the LR in TWs 7, 10 and 14. In contrast to the first IM period, increased *CD4* transcriptions were determined in all cows treated with *MucoCD*-*I* batch B from TW 20, which mainly amounted to > 2 ΔΔCq. *CD8β*, a part of the co-receptor for MHC I molecules, is a characteristic feature of cytotoxic T (T_C_) cells. The *CD8β* expression was unaltered in all groups up to TW 7. During the remaining first IM period, *CD8β* was down-regulated in the treated groups from TW 8, except for the LR in TW 10, and in the Ctr from TW 10. The one-time increase of LR *CD8β* transcripts with the simultaneously depressed HR relative amount of *CD8β* transcripts led to the significant different *CD8β* expressions between them in TW 10. In terms of the treatment with vaccine batch B, the *CD8β* transcription was weakly enhanced in the HR PBL in TW 21 and after TW 25 for the remaining treatment period. Between HR and LR, only one significant variance was found in TW 18. Regarding the low regulated gene *CD19*, coding the B cell-specific co-receptor, the LR stuck out solely with a significant up-regulation of *CD19* in TW 10 compared to HR and Ctr. Contemporaneously with the once injected vaccine batch A, the relative increased transcription of *CD19* in HR PBL was higher than that in PBL of the Ctr group (TW 3). Only one substantially altered regulation of *CD19* was noticed in the second IM period. In TW 31, the transcription of *CD19* in the LR PBL was more than double compared to the associated baseline value. The regulation of the plasma cell typical *CD126*, also known as the cytokine receptor *IL6Rα*, was very strongly changed in the HR group from TW 7 up to TW 14. During this pure nasal IM period, the relative transcription of *CD126* in HR PBL was lowered to roughly three percent of the initial value as measured at the start of the treatment. After the determined higher relative transcript amount of *CD126* in LR PBL towards that of the Ctr up to TW 6, the *CD126* expression of the LR was also reduced for the remaining nasal IM period. However, the extent of this depression in LR PBL was lower than that in HR PBL causing the significant difference between their *CD126* expressions in TW 8. In the Ctr group, the transcription of *CD126* remained unchanged during both treatment periods. With regard to the second IM period, *CD126* was up-regulated in the HR PBLs from TW 21 to TW 29; meanwhile, its relative expressions clearly differed from those of the Ctr group. Additionally, the relative expression of *CD126* in LR PBL exceeded that of the Ctr in TW 25. Among the lymphocyte-related cytokines, *IFNγ*, *TGFβ1, IL2* and *IL10* were investigated to assess the T cell activation state. The expression of the gene coding for *IFNγ*, a cytokine relevant to the activation of macrophages and the Ig class switching, was only slightly impaired by vaccination against *C. difficile* (Fig. 1). This gene was clearly up-regulated in the HR PBL and its relative expressions differed compared to that of the LR at the TWs 2, 4, 6 and 7 in proximity to the once given *MucoCD*-*I* vaccine batch A. At the mid-term of nasal IM, the *IFNγ* transcription peaked once only in the LR PBL. Apart from TW 23, conform developments of the LR and HR relative *IFNγ* expressions in PBL were marked by three simultaneous peaks in the second treatment period with the *MucoCD*-*I* vaccine batch B. The Ctr cows expressed *IFNγ* in PBL primarily steadily. *TGFβ1*, which is known to induce the antibody class switch to IgA production in plasma cells, was found down-regulated in PBL of the Ctr group in TWs 12 and 14. The expressions of *TGFβ1* by the treated groups were not changed for the first 7 weeks of treatment. After depressions up to a quarter of the initial *TGFβ1* levels in PBL of LR and HR in TWs 8 to 10, they were lifted up to the baselines again. Furthermore, the *TGFβ1* starting values of the treated groups in TW 16, marking the second IM period, were significantly raised in TW 27. Immediately after the first treatment with vaccine batch B and additionally in TW 29, the relative *TGFβ1* levels of the HR were significantly superior to that of the Ctr. From TW 8, the IM caused a very strong regulation of the T cell growth factor *IL2*. On this date, undulated expressions of *IL2* in PBL of both treated groups started and lasted during the second vaccination period, too. The Ctr expressed *IL2* in a relatively stable way, but among the treated cows, strong up-regulations by > 4 ΔΔCq of *IL2* in PBL were partly presented by the LR in TWs 8, 18 and 27. On these dates, the relative *IL2* expression levels of the LR differed considerably from those of the HR. The *IL10* expression was analyzed with respect to this cytokine role as an active suppressor of macrophage functions, *inter alia*. The Ctr group showed unchanged *IL10* expressions in PBL over time. Among the treated cows, the transcriptions of *IL10* were weakly depressed several times during the first IM period, mainly by the HR. Consequently, significant differences between the relative *IL10* expression levels in HR and LR PBL existed at the TWs 2 and 10. Within the second IM period, *IL10* was slightly up-regulated in the PBL of both treated groups in TW 21. In addition, the relative *IL10* transcript amounts in PBL varied between Ctr and HR in TWs 23 and 25, and in TW 27 in case of the Ctr and the LR.

### Total and differential cell counts in blood

**Fig. 2:**
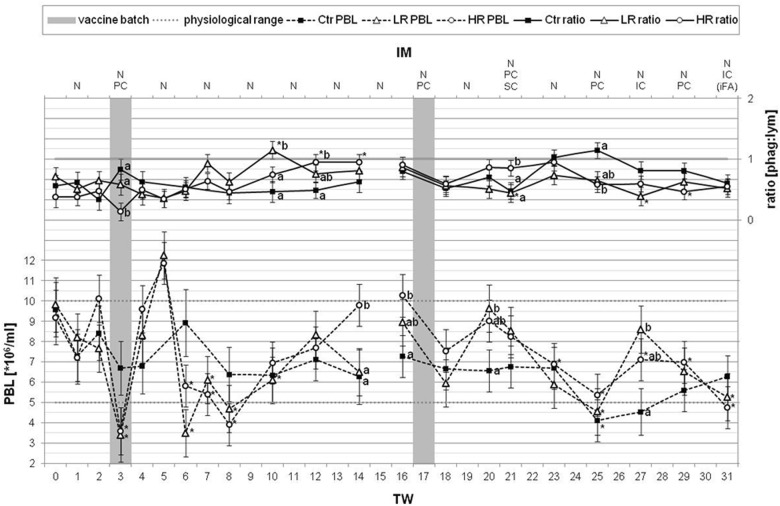
Total PBL contents and the ratios of phagocytes to lymphocytes in blood. On the ordinates, the total PBL contents and the ratios of phagocytes to lymphocytes in blood are depicted as LSM ± SD for the three examined animal groups [(“Ctr” control animals (n = 5), “LR” treated, low responder animals (n = 4), “HR” treated, high responder animals (n = 5)]. The physiological range of PBL (5 up to 10 million leukocytes per milliliter blood) was accented by two gray dotted lines. The 31-week treatment period (TW) is depicted on the lower abscissa. The upper abscissa indicates the different application routes [(nasal (N), percutaneous (PC), subcutaneous (SC) and intracutaneous (IC)] of the vaccines to the TWs with immunization (IM). Only once, incomplete Freund’s adjuvant (iFA) was supplemented to the *MucoCD*-*I* vaccine. The gray bars in TWs 3 and 17 highlight the times of the first uses of the vaccine batches A and B, respectively. During the treatment periods with both vaccine batches and the particular TW before the first use of vaccine batch A or B, substantial differences within the same group are marked by asterisks (*p* < 0.05). Lower case (a, b, c) label evident differences (*p* < 0.05) between the groups at the same dates

**Fig. 3:**
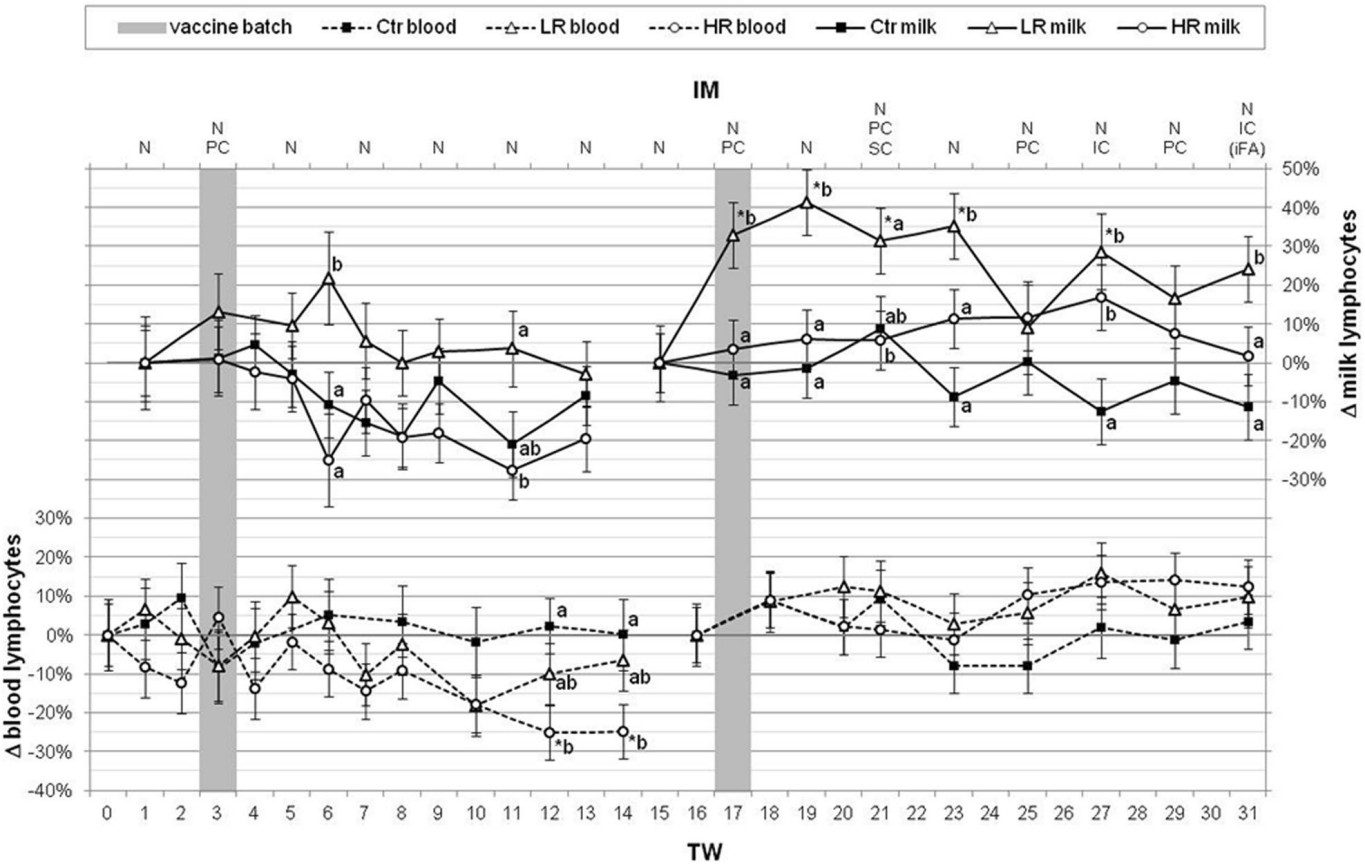
Relative changes of lymphocytes out of milk and blood related to the used *MucoCD*-*I* vaccine batches. The percentage variations of lymphocyte contents per group [(“Ctr” control animals (n = 5), “LR” treated, low responder animals (n = 4), “HR” treated, high responder animals (n = 5)] and per milk and blood are represented as LSM ± SD on the vertical axes. The comparative indices are the treatment weeks (TW) before the first-time application of the vaccine batches A or B, respectively. The upper abscissa shows the used routes for administration of the vaccine [nasal (N), percutaneous (PC), subcutaneous (SC) and intracutaneous (IC)] marking the dates of immunization (IM), too. The *MucoCD*-*I* was given only once plus incomplete Freund’s adjuvant (iFA) in TW 31. In TWs 3 and TW 17, the primal injections of the vaccine batches A or B are accented by the gray bars. Deviated by group, the asterisks (*p* < 0.05) indicate considerable differences relating to the dates immediately before the first use of each vaccine batch. The significant variations (*p* < 0.05) between the groups at the same times are characterized by small letters (a, b, c)

The total and differential cell numbers were assessed referring to the physiological ranges of bovine PBL [25]. Typically, bovine blood contains five to ten million PBL per milliliter. Comparing within the groups, control (Ctr), LR and HR, the average PBL amounts per milliliter blood were similar with 6.8 ± 1.3 (Ctr), 7.1 ± 2.6 (LR) or 7.5 ± 2.4 (HR) million cells for the examined treatment period of 31 weeks. During the course of treatment, the graphs for the PBL concentrations of both treated groups developed predominantly in a similar manner (Fig. 2). After a singular IM with *MucoCD*-*I* batch A, the LR and the HR cows blood included the significantly lowest and highest PBL concentrations in TW 3 and TW 5, respectively, and furthermore beyond the normal range. One more time, the LR PBL count fell considerably below the minimum threshold in TW 6. With some delay, the PBL number of the HR group achieved a second low with 3.9 ± 1.0 million cells ml^−1^ in TW 8. During the following exclusively nasal IM, the continuous increase of the HR PBL contents led to a distinctively higher value, amounting to more than nine million cells in TW 14. In the second vaccination period starting in TW 17, the treated cows showed a trend to reduce PBL concentrations to slightly below the physiological minimum level for the LR in TW 25 and for the HR in TW 31. The Ctr group stood out with considerable few PBL concentrations of unknown cause in TW 25 and TW 27. The relative distributions of the single leukocyte populations depending on different times of measurement are summarized in Additional file 4: Table S4. In principle, the proportion of lymphocytes in bovine blood varies between 45 and 65%, and the subjacent range of PMN is between 25 and 45% [25]. These are the dominant cell types and, by taking also the monocytes into account for up to 6%, the normal ratio of phagocytes to lymphocytes is faintly < 1 in bovine blood. Evaluating significantly positive divergences of this ratio during the IM period, 1.2 ± 0.2 was determined for the LR group in TW 10 (Fig. 2). Additionally, out of the standard range, the same level of this ratio was found for the Ctr group in TW 25. Focusing on the development of the blood lymphocytes (Fig. 3), the first IM period with the once injected *MucoCD*-*I* batch A followed by the solely nasal IM, a clear reduction of the HR lymphocyte concentrations in blood emerged in TW 12 and TW 14. However, the delta values of lymphocytes in blood of LR and Ctr progressed steadily further on. During the treatment period with *MucoCD*-*I* batch B, there were no noticeable alterations of the lymphocyte contents in blood.

### bMEC-related genes in SCC

In terms of the systematic triggering of the cows IS by vaccination against *C. difficile*, the adjustments of the bMEC’ related *PIGR* and *FcRN* were analyzed as the key receptors for epithelial transports of IgA and IgG, respectively. *PIGR* was graded as strong regulated due to its increased expression by > 3 ΔΔCq in HR SCC in TW 21. Differently altered expression levels of *PIGR* existed only once in each IM period, between LR and Ctr in TW 6 and between HR and Ctr in TW 17. The expression of *FcRN* was not affected by the treatment in any group. The complement system, represented by *C1QA* and *C3*, was barely impaired by the vaccination procedure. No relative expression changes of *C1QA* were determined during the treatment. The gene *C1QA* codes for the recognition protein C1q, a part of the complement component C1 that initiates the classical pathway of complement activation by antigens. Regarding the alternative pathway that can be initiated by cleavage of the complement component C3, the gene *C3* was weakly regulated. During the first IM period, the relative *C3* expression in SCC of LR more than doubled, evoking also the significant difference to that of the Ctr in TW 5. Three TWs later, in TW 8, the same relative *C3* transcript amounts of LR and Ctr were, on average, significantly higher than those of the HR. Following treatment with *MucoCD*-*I* batch B, the LR *C3* was expressed less compared to the other groups in TW 27. A selection of chemokines classified as bMEC’ related was analyzed to verify the relevance of bMEC for the recruitment of leukocytes to the mammary gland in response to the repeated vaccination against *C. difficile*. Among the genes that encode the chemokines, the expression of *CCL5* was slightly interfered by the vaccination, which was significantly down-regulated in HR compared to Ctr in TW 5. *CCL20* was found strongly regulated during treatment against *C. difficile*. In the course of the first IM period, the relative expression levels of *CCL20* differed to their baselines for the LR in TW 7 and for the Ctr in TW 9. Concerning heterogeneous regulations of *CCL20* between the groups, two gaps were determined between HR and Ctr in TWs 3 and 9, whereas during the second IM period, the down-regulated transcriptions of *CCL20* in SCC of HR compared to the up-regulated ones of the Ctr varied on each date of analysis. Without developing divergent expression levels of *CCL20* in SCCs to the belonging initial value in TW 15, the LR produced lower relative transcript amounts of this chemokine than the Ctr on four dates. In TW 27, the date of the first IC application of the vaccine batch B stood out with only one-time different expression levels of LR and HR. At no time of treatment, *CCL28* was altered to its baselines in SCCs of any group. Nevertheless, the regulation of CCL28 was assessed to be weak due to the proven differences between groups. Variations between the transcription levels of *CCL28* in SCCs per group existed only during the pure nasal IM period. Apart from TW 8, the calculated transcript amounts for the LR cows were significantly higher to that for the Ctr cows on every date of measurement beginning with TW 5. The HR *CCL28* expression was markedly down-regulated compared to the LR in TW 9. Likewise, *CXCL3* was graded as slightly regulated. While no alterations of the gene expression could be identified in any group during the first IM period, the down-regulation of *CXCL3*, as found in SCCs of the HR group by vaccination with *MucoCD*-*I* batch B, was decisive for the classification of this gene. Significant variations between the relative expression profiles of *CXCL3* in SCCs of the treated groups and the Ctr group existed predominantly on the same dates. No changed regulations of *CXCL5* were noticed as induced by the vaccination against *C. difficile.* Regarding the chemokine *CXCL8*, a weak regulation was shown because of a single significant down-regulation between the treated groups and the Ctr group in TW 23. Within the analyzed antimicrobial factors as produced by bMEC, the gene coded for lactoperoxidase, *LPO*, was solely found as strongly regulated. The *LPO* expression patterns of all groups had not been changed towards the associated baselines in TWs 1 and 16, but two dates with discrepancies between treated and Ctr cows were found. At the beginning of the pure nasal IM, the relative *LPO* transcript amounts of the treated groups exceeded that of the Ctr group in TW 5. One TW later, the ∆∆Cq value of 3.4 for the LR *LPO* transcription was significantly above that for the Ctr group. The treatment interfered only slightly with additional genes belonging to the division of antimicrobials like lactoferrin (*LF*), lysozyme (*LYZ1*) and tracheal antimicrobial peptide (*TAP*). Following vaccination with *MucoCD*-*I* batch A, *LF* was inversely regulated in the SCCs of the treated groups. The up-regulation of *LF* in the LR group and its simultaneous down-regulation in the HR group resulted in significant differences between them in TWs 7–9 and 13. The LR *LF* transcription was also intensified towards the Ctr in TWs 5–7. At the first application of *MucoCD*-*I* batch B, the HR *LF* expression level exceeded significantly compared to that of the Ctr (TW 17). In context with the triple-vaccination on the nasal, PC and SC route, *LF* was clearly expressed more in the treated cows compared to the baselines and to the Ctr in the TWs 21 and 23. In mid-term of the first IM period, sharply reduced transcriptions of *LYZ1* were noticed for the Ctr cows on several dates and for the HR cows only in TW 11. Different regulations of *LYZ1* between the groups were not determined during the period to be analyzed. Related to the application of *MucoCD*-*I* batch A, the apparently diminished *TAP* expressions in the SCCs of both treated groups led to the proven variations towards the *TAP* expression levels in Ctr SCCs in TWs 3 and 5. Nevertheless, those of the treated groups were neither different to the respective initial values on these dates nor to other measurement points during the complete treatment period. Finally, at the end of treatment with vaccine batch B, a higher relative transcript amount of *TAP* was found in LR than in Ctr cows.

### Phagocyte-related genes in SCC

Among both analyzed genes, which encode complement receptors, *C3AR1* was assessed to be regulated on a minor level in SCCs than in *C5AR1*. In the course of the complete treatment period, no group showed considerable alterations of *C3AR1* expressions towards the belonging initial values. But always towards the end of both vaccination periods, the expression profiles of the treated groups differed clearly from those of the Ctr group. For the HR and LR, the first IM period finished with expression levels of *C3AR1* showing an upward tendency. Conversely, their expression levels of *C3AR1* tended to be reduced at the end of the second IM period. During both IM periods, the developments of the *C5AR1* transcriptions in SCCs of the treated groups towards the Ctr group were similar to those of the *C3AR1* transcriptions. However, the relative transcript amounts of *C5AR1* in the SCCs of the treated groups exceeded considerably towards the baselines when finishing the first IM period. That was also the case for the development of *CXCR2* transcripts in the HR SCCs. Generally, this chemokine receptor was regulated on a low level. In contrast to the Ctr cows, CXCR2 was significantly higher expressed by the HR in TW 13 and significantly lower expressed by HR and LR in TW 23. *TLR2* belonged to the category of weakly regulated genes by the treatment. During the first IM period, only in the HR group, a single up-regulation of *TLR2* towards the baseline was determined in TW 8. Comparing the relative expression levels of *TLR2* in SCCs between the three groups, those of the HR were markedly higher than those of the Ctr in TWs 9 and 11. Furthermore, the relative HR transcript amounts of *TLR2* also topped those of the LR in TW 11. At mid-term of the vaccination with *MucoCD*-*I* batch B, the *TLR2* transcriptions in SCCs of both treated groups were significantly reduced compared to the Ctr group. The gene for the specific surface determinant of monocytes and macrophages, *CD163*, was graded as non-regulated by the vaccination against *C. difficile*. Concerning the investigated gene regulations of phagocyte-specific cytokines, *TNFα* was only determined to be strongly interfered by the treatment. The steadily increasing *TNFα* transcription in SCCs of the Ctr group, as proven during the second IM period, was crucial for this classification. Two weeks after PC injection of vaccine batch A, the reduced transcription of *TNFα* by more than 80% in the SCC of HR was considerable towards the baseline, and, in addition, by comparing it with the *TNFα* transcription of the Ctr group (TW 5). Similarly, the *TNFα* expression in the HR SCC was depressed in TW 13. During the second IM period, the *TNFα* expression profiles of the treated groups developed similarly. They were shaped by a reduction up to TW 21 followed by an increase that reached their initial values at the last point of measurement in TW 31. Continuously, *TNFα* transcript amounts in the treated groups were significantly lower than those of the Ctr cows. The interleukins, *IL1β*, *IL6* and *IL12β*, were graded as slightly regulated in SCCs during 31 TWs. After the *MucoCD*-*I* vaccine batch A was administered, the LR *IL1β* was exclusively markedly lower expressed than the Ctr one in TW 5. Regarding the second period of vaccination, both *IL1β* transcriptions in the SCCs of the treated groups were significantly diminished as compared with that of the Ctr in TW 23. At the following points to be analyzed, the *IL1β* transcriptions in SCCs of LR were steadily multiplied and those of HR were constantly reduced resulting in significant differences between HR and LR as well as between HR and Ctr in TW 27. In context with the once given vaccine batch A, *IL6* was clearly down-regulated in the HR SCC in TW 5. On the same date, this gene was also differently expressed between the HR and LR cows. In TW 13, the *IL6* expression in the Ctr SCC was noticed by its significant decrease towards the associated baseline value. During the second IM period, the development of the treated cows *IL6* expression profiles was characterized by initially reduced transcriptions of *IL6* compared with the Ctr cows. These were significant in TWs 21, 23, 27 (only HR) and 29. Then, the *IL6* expressions in SCCs of HR and LR returned to the baseline levels in the last TW. In case of the examined *IL12β* expressions in SCCs of the treated and the Ctr groups, a significant gap existed between them during the first IM period in TW 9. Within the treatment period that used vaccine batch B, the increasing transcription of *IL12β* in SCCs of the Ctr resulted in their clearly higher transcript amounts as measured by those of the treated groups. The significant variances between the groups were supported by a similar trend towards reduced *IL12β* transcriptions of all treated cows up to TW 23.

### Lymphocyte-related genes in SCC

**Fig. 4:**
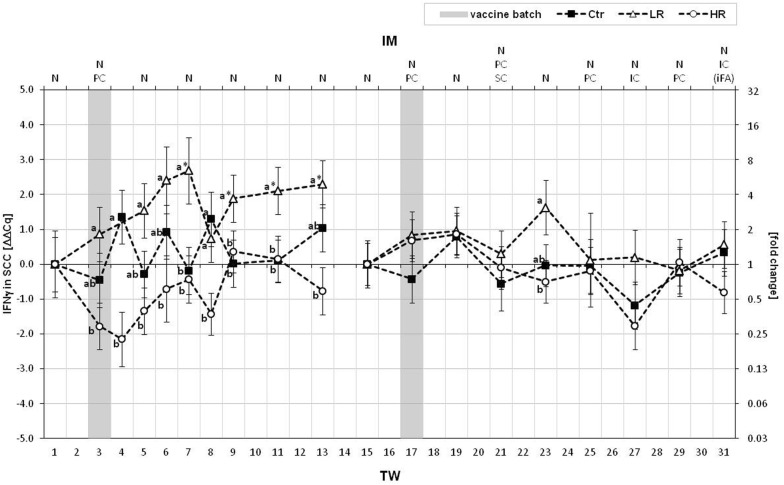
IFNγ gene expression in SCC. The Y-axis shows the ΔΔCq values of IFNγ as LSM ± SD and the corresponding fold changes for the control (“Ctr”, n = 5) and the treated animal groups (“LR”, low responder cows, n = 4; “HR”, high responder cows n = 5). The 31-week treatment period (TW) is depicted on the lower X-axis. The immunization (IM) specified by the routes of vaccine application [nasal (N), percutaneous (PC), subcutaneous (SC) and intracutaneous (IC)] per TW is illustrated by the upper X-axis. Incomplete Freund’s adjuvant (iFA) was only once part of the *MucoCD*-*I* vaccine in TW 31. The gray bars in TWs 3 and 17 highlight the times of the first uses of vaccine batches A and B, respectively. During the treatment periods with both vaccine batches and the particular TW before the first use of vaccine batch A or B, substantial differences within the same group are marked by asterisks (*p* < 0.05). Lower case (a, b, c) indicate evident differences (*p* < 0.05) between the groups on the same dates

Considering the adaptive IS, the T cell population marker *CD3δ* was only weakly impaired in SCCs by the repeated vaccinations against *C. difficile*. In context with the once injected vaccine batch A, the relative expression of *CD3δ* of the LR was found changed by roughly 2 ∆∆Cq compared to the other groups in TWs 3 and 6. Within the second vaccination period, the differences in *CD3δ* expression levels between the treated and the Ctr groups were mainly attributed to their inverse courses of *CD3δ* regulation. *CD4*, tagging the T_H_ cell populations, was very strongly up-regulated in the SCC of LR in TW 3. On this date, the LR transcription of *CD4* amounted to 3 ∆∆Cq, which was considerably different from HR and Ctr. The extent of this peak led to the assessment of *CD4* being strongly influenced by the treatment. From mid-term of treatment with *MucoCD*-*I* batch B, two gaps between the developments of the *CD4* expressions by the treated cows and the Ctr cows became obvious. The relative *CD4* transcript amount in the SCC of the HR group was significantly lower than the Ctr one in TW 23, and this was also the case between the LR and the Ctr in TW 29. Regarding *CD8β*, marking the T_C_ cells, this gene was graded as slightly interfered by the vaccination on numerous dates to be analyzed. In SCCs of LR and Ctr, their *CD8β* expressions developed contrarily in TWs 9 and 11 within the solely nasal IM period. Additionally, the HR and Ctr expression levels of *CD8β* differed in the SCCs in TW 11. During the second IM period, *CD8β* was markedly lower expressed in the HR compared to the Ctr group in TW 23. The transcription of the B cell receptor *CD19* was slightly influenced by the vaccination as measured in SCCs. The inconsistent development of the Ctr *CD19* expression for the first TWs was decisive for the variations from the treated groups. Following the treatment, *CD38* marking activated B and T cells within the SCCs, was found to be weakly regulated. Within the groups, no different *CD38* expressions in opposition to the baselines were noticed. Between the groups, two gaps were determined in the course of the first IM period. The first one was present between the relative *CD38* transcriptions of the HR and LR in TW 3, and the second one was determined between those of the HR and Ctr in TW 9. The surface determinant of activated B cells and plasma cells *CD126* was characterized by a commonly tenuous regulation related to its expression by SCCs of all groups. Only the LR group showed the trend to a decreasing expression of *CD126* between TW 6 and TW 8, which was sufficient to achieve distinct differences towards the Ctr group. Additionally, the course of the LR *CD126* expression in SCCs led also to reduced transcript amounts of *CD126* versus the HR in TW 8. The generated transcript profiles of *CCR6*, the counterpart to *CCL28,* were similar to one another for both treated groups. Apart from the single disparity between the *CCR6* regulations of the HR and the Ctr in TW 5, no further alterations of the generally classified as weak *CCR6* regulation were found within and between the groups during the first IM period. In the course of the second IM period, the LR differed from the Ctr more hesitantly than the HR from the Ctr and the HR *CCR6* was significantly up-regulated from TW 25 up to TW 31. *IFNγ* and *IL10*, which, *inter alia*, are produced by T lymphocytes, were dissimilarly regulated due to the vaccination procedures. A weak altered expression characterized *IFNγ*, whereas the expression of *IL10* was classified as strongly changed. Comparing the treated groups, inversely progressed *IFNγ* expressions in the SCCs were detected from the beginning of treatment (Fig. 4). In these first TWs, the LR’s transcription of *IFNγ* was increased by partly > 2 ∆∆Cq in TWs 3, 5–13, while that of the HR was noticeably depressed towards the LR all the time except for TW 9. In the course of treatment with the *MucoCD*-*I* vaccine batch B and, in contrast to the first IM period, the relative expressions of *IFNγ* in SCCs of the LR and the HR varied only once in TW 23. The Ctr cows expressed *IFNγ* in SCCs primarily steady during the 31 TWs. Differently developed *IFNγ* expressions between the Ctr and the treated groups existed only in TWs 4, 7, 8 and 11 during the first IM period. Unlike the Ctr, the inhibitor of important immune functions of the innate IS, *IL10*, showed to be more down-regulated in the SCCs of the treated cows following the IM with *MucoCD*-*I* vaccine batch A on several dates. However, only in TW 13 was the depressed expression of *IL10* in the SCC of the LR heavy enough to differ clearly from the baseline. The levels of *IL10* expressions in the SCCs varied from TW 15 neither within nor between the groups.

### Total and differential cell counts in milk

**Fig. 5:**
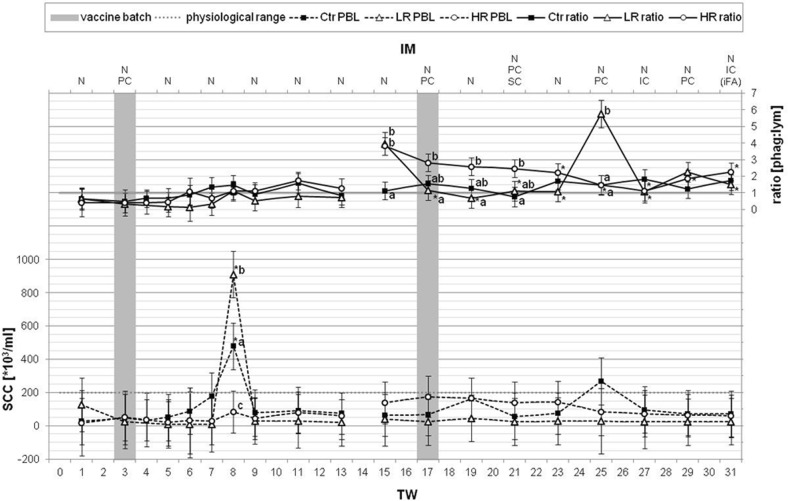
Total SCC in milk and the associated ratio of phagocytes to lymphocytes. Within 31 treatment weeks (TW), the recorded total SCC in milk are shown in groups [“Ctr” control animals (n = 5), “LR” treated, low responder animals (n = 4), “HR” treated, high responder animals (n = 5)] as LSM ± SD on the lower vertical axis. The gray dotted line at 200 thousand cells per milliliter marks the commonly used cut-off value for normal SCC. The upper line chart depicts the ratio of phagocytes to lymphocytes in milk. The routes of vaccination [nasal (N), percutaneous (PC), subcutaneous (SC) and intracutaneous (IC), incomplete Freund’s adjuvant (iFA)] are presented on the upper abscissa on the immunization (IM) dates. Two gray bars represent the IM times using the *MucoCD*-*I* vaccine batches A or B. The asterisks (*p* < 0.05) indicate the inner-group deviations in relation to the dates before the initial uses of both *MucoCD*-*I* vaccine batches. The significant differences (*p* < 0.05) between groups on the same dates are marked by lower case letters (a, b, c)

Generally, dairy farming accepts the cut-off value of 200 thousand cells per milliliter bovine milk for assessing udder health [31, 32]. When monitoring the SCC in milk (Fig. 5) over the IM period, this practice-related upper limit was exceeded by a multiple only at one time. In TW 8, the LR and Ctr groups protruded with high SCCs amounting to 910.1 ± 139.8 and 479.3 ± 139.8 thousand cells per milliliter milk, respectively. Those group-related SCC levels were dominated by data of single cows. An acute mastitis was demonstrated for cow LR-2 causing more than 3.5 million cells per milliliter milk, whereas the SCCs of the other LR cows accounted for around 15 thousand cells per milliliter milk. The source of the striking Ctr cow SCC with nearly 2 million cells per milliliter milk was not found. Its udder health seemed to be unimpaired. All other cows of the Ctr group showed a SCC of 70 thousand cells per milliliter milk, on average, in TW 8. The results of the DCC in milk are compiled in detail in Additional file 5: Table S5. The development of the lymphocyte proportions in milk was investigated in relation to the date before the first use of each *MucoCD*-I vaccine batch (Fig. 3). The untreated Ctr showed a clear decrease of these immune cells unlike the homeostatically balanced lymphocyte contents in blood during both IM periods. In LR milk, the level of the lymphocytes grew by 20% up to TW 6 and declined towards the baseline in TW 13. Similarly to the Ctr, the lymphocyte contents in milk of HR declined reaching a low of − 30% in TW 12. After the application of vaccine batch B in TW 17, the development of the lymphocyte proportions in milk of LR was strongly influenced by the extraordinarily low baseline value in TW 15. Ruling out this effect, the lymphocyte levels in the milk of the LR group also showed a declining trend based on TW 19. In comparison, the lymphocyte contents of the HR milk appeared slightly volatile by maximal + 17% to the date of the firstly IC given *MucoCD*-*I* and returned to the initial level in the last TW. Emphasizing the calculated ratios of phagocytes to lymphocytes per group (Fig. 5), they developed predominantly uniformly within TW 1 up to TW 13. During this first IM period, a slight increase of this ratio from 0.6 up to 0.9 was equally noticed for all three groups. This development continued in case of the Ctr, even in the remaining TWs. General phagocyte growth was mainly characterized by the increasing percentages of the PMNs in proportion to the lymphocytes. Notwithstanding the varying development of these cell populations in the treated groups within TW 15 and TW 25, the ratios of phagocytes to lymphocytes of all groups averaged 1.8. The extraordinarily high values of this ratio of about 3.9 in each treated group were due to an encounter of maximal PMN and low lymphocyte concentrations in milk in TW 15. Likewise, the LR milk contained a large share of phagocytes due to an individual PMN count of around 90% in TW 25. The physical check of the responsible LR cow revealed an unremarkable status of health. The described extreme levels of the phagocytes to lymphocytes ratios in milk were not assessed as to be induced by the vaccination regime, whereas the quotients formed by phagocytes and lymphocytes differed distinctively between the LR and HR after the first injection of vaccine batch B for the following 4 weeks.

## Discussion

### Clostridium difficile specific IgA in milk

The specific antibody response against *C. difficile*, more precisely the level of anti-*C. difficile* IgA in milk, was the decisive criterion for assessing the immune reactivity of the treated cows [21]. In search of indications that primarily explained their different responsiveness to the repeated challenging with *C. difficile*, the outcomes of this study were appraised hereinafter. For this, in particular, the first three TWs were of interest upon receiving the *MucoCD*-*I* batches A and B for the first time. For these dates, the main gaps between the specific antibody responses of both groups were ascertained amounting to a threefold (batch A) or sevenfold (batch B) varying anti-*C. difficile* IgA content in milk [21]. In case of the LR, the amount of anti-*C. difficile* specific IgA in milk exceeded only once the associated initial value in TW 5. But the lastingly increased production level of *C. difficile* specific IgA in HR milk of about at least a triple upon TW 3 remained unaffected by this single peak value in LR milk [21].

### Gene expression profiling in PBL

Within the scope of the gene expression analysis, an impression of alterations on the molecular biological level induced by the repeatedly administered inoculation was received. Commencing with the genes investigated in PBL and related to the innate IS, the two-part vaccination schedule had scarcely interfered with the expression of the phagocytes characterizing surface determinants. Even though, in response to the poorly tolerated vaccine batch A, the first phase of the biological leukocyte curve, as shown by clearly reduced PBL counts (Fig. 2), were also determined by the depressed expression of the blood phagocyte receptors (*C5aR1, CXCR2, TLR2, CD163*) in TW 4. In part, the down-regulation persisted for these genes throughout the remaining first IM period like for HR and LR *CXCR2* and for the HR *CD163*. Although this might hint at a possibly induced migration of phagocytes leaving the circulation, the phenotypic confirmation of this assumption by DCC was not given. In response to the first application of *MucoCD*-*I* batch B (TW 18), the HR cows responded more sensitively than the LR, as measured by increased *CXCR2* expression in HR PBL. Simultaneously increased PMN numbers in HR blood were not determined, even though *CXCR2* is predominantly expressed by PMN [33]. Possibly, the *CXCR2* up-regulation in HR PBL was necessary in order to clear the corresponding ligands that were released at the vaccination site, and to restore their homeostasis [34]. Tracing the injected *C. difficile* antigens by complement during the second IM period seemed to be of subordinate importance assessed by means of the phagocyte-related complement receptors. Likewise, the *C. difficile* antigen detection by the *TLR2*, which is of prime importance for the recognition of gram-positive cell wand components, appeared to be on a low level [35]. Nonetheless, *TLR2* was equally up-regulated within the PBL of the treated groups following the triple vaccination in TW 21. Conceivably, the dendritic cells were involved predominantly in the sensing of the *C. difficile* antigens subsequent to its inoculations. These APCs are located within the peripheral tissue out of the blood circulation and they are the main initiator and modulator of the immune response [36]. The examined chemokines, which are known to be primarily emitted by monocytes/macrophages, were differently expressed in the HR and LR PBL, especially in relation to the application of *MucoCD*-*I* batch A. During the first IM period, the chemokine profile of the HR was characterized by more pronounced down-regulations of *IL1β* and *IL6* and a partly stronger up-regulation of *CXCL8* in contrast to the LR. For the HR, this might mean that vasodilation for recall of defined leukocyte populations, like PMNs and effector *CD8* positive T cells, was promoted, but that no further pro-inflammatory signaling was supported [28, 37], whereas the LR showed a strong up-regulation of *IL12β*, the prerequisite for the T_H_1 cell response [38]. The second IM period indicated more consistent expressions of the chemokines by both treated groups. Uniformly, following the triple vaccination in TW 21, *IL1β* was down-regulated once in the short term, *IL12β* was down-regulated in the long term, and *IL6* was permanently up-regulated in PBL of HR and LR. Thus, the triple vaccination could have caused the switch to a T_H_2 immune response driving the antibody production by plasma cells [38]. Owing to the chemokines importance to orchestrate the lymphocytes, their different expression patterns might have been one of the crucial determinants for the different reactions of the treated groups to the vaccinations against *C. difficile.* Based on the partly strong regulation of lymphocyte specific receptors, like *CD3δ* and *CD126,* it can be assumed that the associated cell proportions of T cells and activated B cells were equally influenced by the treatment. While *CD3δ* was not differently expressed between HR and LR in the course of the first IM period, the T cell subpopulations marking surface determinants, *CD4* and *CD8β*, were regulated in a clearly different way between them. Following three and four TWs after the treatment with *MucoCD*-*I* batch A, *CD4* and *CD8β* positive T cells were down-regulated more sustainably in HR than in LR. This could be caused by their differing profiles of chemokines on this date. Exemplarily, the previously enhanced *CXCL8* expression levels could have activated the withdrawal of *CD8β* positive cells out of the blood stream [37]. Additionally, *IL6* was contemporaneously down-regulated with the *CD4* positive T cell subset. The primarily lower depressed expression of *CD4* and *CD8β* positive T cells in LR than in HR could be associated with the more intense increase of *IL12β* transcripts, as *IL12β* supports cellular immunity [38]. The pronounced up-regulation of the T cell growth factor *IL2* in LR PBL in TW 8 as well as the missing up-regulation of the inhibitory active *IL10* could also have contributed to that [39]. *IL10* was partly down-regulated in the HR PBL, whereby the chemotactic effect of possibly secreted *CXCL8* would not have been hampered. Concerning the B cells, the extent of the drastic down-regulation of the *IL6* recipient, *CD126*, in LR and HR did not reflect the corresponding *IL6* expression patterns. With a view on the second IM period, similarly to the interleukins, the expressions of the different lymphocyte markers were mainly uniformly developed by HR and LR during this treatment period, too. Obviously, the triple vaccination (TW 21) could have caused a very strong decrease of *CD3δ* positive T cells paired with the growth of the associated subpopulation, the *CD4* positive T cells, whereas the level of *CD8β* positive T cells seemed not to be affected by this treatment. The TCR down-modulation upon ligation with MHC:peptide complexes has been demonstrated to be important in T cell activation [40]. The T cell activation through the TCR lead to the subsequently de novo synthesis of *IL2* [41]. The presumed proliferation of *CD4* positive T cells in TW 21 might be triggered by the simultaneously increased release of *IL2* and *IL6*. The likely withdrawal of *IL12* could indicate the switch of *CD4* T cell differentiation into T_H_2-like cells. Their maturation is probably activated by *IL6* [38]. Finally, the extent of the possible T_H_2 immune response was regulated by probably secreted *IL10*, because the associated transcripts were multiplied in the treated groups from TW 21, too. *IL10* participates in inhibiting *IL12* and *IFNγ* secretions [39]. Although *IFNγ* and *IL2* are generally classified as typical T_H_1 cytokines, both were proven to be up-regulated at the same time during treatment with vaccine batch B. This is remarkable considering the clear down-regulation of *IL12* at these points in time, which is deemed to be a potent inducer of *IFNγ* in T cells [42]. On the other hand, a positive feedback mechanism interconnects both cytokines in inflammation [39], 42]. However, the causal link between *IL12* and *IFNγ* could not even be fully established for the first treatment period, as *IL12* and *IFNγ* were predominantly inverse regulated following the anti-*C. difficile* vaccinations during this period. It is known that discrepancies in the T_H_1/T_H_2 hypothesis exist [38, 39]. The paradigm of type 1 and type 2 immune responses was established with laboratory rodents and it is not entirely applicable to large ruminants [38, 43]. Considering the effect of *IFNγ* to promote the antigen recognition and the Ig class switching in favor of IgG, this cytokine seemed to play an important role in the two-part responsiveness of the treated cows [43, 44]. As opposed to the LR, the HR showed stronger induced *IFNγ* expressions by the treatment on four dates during the first IM period and to a further date following treatment with vaccine batch B (Fig. 1). Generally, *IFNγ* supports intense phagocytic activity and only a weak humoral antibody response [39]. However, the DCC did not confirm enhanced concentrations of phagocytes in the TWs with up-regulation of *IFNγ* in the treated groups. In addition, the measurement of IgG concentrations in blood and milk were not part of the study at hand, so the possible effects of *IFNγ* were not verifiable. *TGFβ1* is referred to as an important regulator of mucosal immunity and as the co-responsible cytokine for induction of the antibody class switch to IgA [39, 44]. Regarding the examined anti-*C. difficile* IgA response, *TGFβ1* was not differently regulated between the treated groups. Also, in the crucial TWs of significantly increased IgA concentrations in HR milk, the *TGFβ1* expression did not differ between HR and Ctr in PBL. The examination of the local IS of the mammary gland as measured by gene regulations in SCC revealed only few differences between the treated groups in response to the vaccination. The different anti-*C. difficile* IgA production in milk of LR and HR did not seem to be influenced by different expression levels of the epithelial IgA receptor. The *PIGR* expression in the lactating mammary gland is assumed to be primarily controllable by steroid hormones rather than by immunoregulatory factors [45, 46]. Concerning the complement system, the complement activation product, C3a, is described as an inflammatory modulator [47]. The differently regulated complement component *C3* between HR and the other groups, LR and Ctr, in TW 8 has to be seen within the context of the excessive SCC values of LR and Ctr to this date (Fig. 5). Inflated SCC in milk usually indicates an inflammation, and for example, intramammary infections might cause increased *C3* expressions by bMECs [48]. In contrast to the HR and partly to the Ctr, the antimicrobial peptide *LF* and the chemokine *CCL28* were up-regulated in the LR group around TW 8, too. Possibly, both immunological factors had contributed to overcome an impending udder infection of the LR. Considering their up-regulation from TW 5 towards unchanged expressions of *LF* and *CCL28* in the Ctr, it is also likely that the vaccination increased their transcription in LR. Besides the direct antibacterial activity of *LF*, it operates cytokine-like as an “alarm”, summoning assistance by various leukocytes [49]. *CCL28* possesses also antimicrobial activities, but the typical feature attributed to this chemokine is to accumulate lymphocytes, especially IgA plasma cells, in mucosal tissues [50]. Thus, *CCL28* is designated as key regulator of B lymphocyte migration and retention in the mammary gland [51]. In all treated cows inoculated with *MucoCD*-*I* batch A, *CCL28* was up-regulated between TWs 5 and 7, supposedly to utilize the *CCL28* functions. The increased expression of *LPO* in the treated cows within the same period could have been precautionary, as this host defense peptide should participate to resolve the potentially microbial threat for the mammary gland by vaccination due to its bacteriostatic and bactericidal properties [52].

### Total and differential cell counts in blood

Concerning the immune cell counts, their total numbers in blood revealed no disparities between LR and HR (Fig. 2) related to the vaccination program. Nonetheless, the PBL counts of all treated cows were definitely impaired by the continuous vaccinations, and they exceed uniformly their physiological range following the once PC administered *MucoCD*-*I* batch A (Fig. 2). In connection with the injection of this poorly tolerated vaccine, the PBL counts of the treated cows developed similarly to Schilling’s biological leukocyte curve as being characteristic of bodily injuries caused by infections or toxins [25]. The PBL falls noticeably for all groups in TW 25 and cannot be exclusively explained by the vaccination schedule because the Ctr group was affected more particularly. An error in the PBL measurements up to this date was excluded, as the Ctr group was also below the physiological range of PBL in TW 27. The blood sampling date was in the early spring and it was assumed that health monitoring did not perceive the effect of a seasonal viral infection.

Regarding the DCC, the development of the lymphocyte parts in blood was primarily investigated in relation to the associated baselines for each IM period and compared to the phagocytes contained therein. Lymphocytes perform the specific immune response to an antigen, and in the narrower sense, they control antibody production [53]. Mucosal immunization, in particular, boosts lymphocyte migration following their activation by an antigen in the draining LNs next to the vaccination site. Afterwards, the activated lymphocytes colonize the related mucosal tissues via the lymph, and finally, via the blood circulation [54]. Thus, the apparent differences between the *C. difficile* specific IgA levels in the treated groups could be caused by crucial variations between lymphocyte numbers. Additionally, because every vaccine itself and the vaccination procedure act as immune stressors, the phagocytes to lymphocytes ratio was evaluated, which is frequently reported as an indicator of physiological stress in farm animals [55]. After the first vaccination with *MucoCD*-*I* batch A, the DCC in the blood of the HR group was immediately dominated by especially low-diameter lymphocytes in contrast to the LR and control groups, as demonstrated by the ratio of phagocytes to lymphocytes (Fig. 2, Additional file 4: Table S4). Nevertheless, the lymphocyte percentage of the HR remained unchanged compared to the baseline in TW 3 (Fig. 3). However, the apparently increased young blood lymphocytes, together with the diminished total PBL content, led to the assumption that a higher proportion of these effector cells of the adaptive IS might have been transported to the vaccination site of the HR cows on this date. The atypical ratio of phagocytes to lymphocytes > 1 in LR blood in TW 10 could not be explained to be caused by any confrontation of the body with the *C. difficile* antigens. This appraisal was assured by noticing a similar event in the Ctr group in TW 25. The discrepancy between the ratios of phagocytes to lymphocytes within the PBL of the treated groups in TW 21 was evoked by the significant increased percentage of phagocytes in the HR DCC, which depended mainly on the enhanced proportion of monocytes in contrast to the LR (Fig. 2, Additional file 4: Table S4). A modest monocytosis can be a sign of physiological stress [56], and possibly, the IS of the HR had reacted more sensitively than the LR IS by an additional SC confrontation with the antigen for the first time.

Contrary to the one-time administered vaccine batch A, the systemically re-exposure to *C. difficile* by vaccine batch B did not initially lead to increased lymphocyte percentages in the blood of HR cows, whereas their specific antibody production against *C. difficile* was clearly enhanced between TWs 17 and 21 (Fig. 3, Additional file 4: Table S4) [21]. Therefore, a boost of the existing *C. difficile* specific T and B memory cells can be assumed, causing shifts of subtypes within the relative proportion of the blood lymphocytes but without impacting the size of the relative proportion itself. The continual re-stimulation of B cells to proliferate and differentiate into antibody producing cells by intermittent re-exposure to the pathogen, as is the case in vaccination, promotes long-term antibody production, and usually the magnitude of antibody production depends on the lifespan of plasma cells [57]. Under steady-state conditions in vivo, one-time vaccination triggers initially the rise of antigen specific serum antibody levels, circulating plasma cells and activated memory B cells, but this correlation can be lost after a boost, which causes only a pronounced increase in antibody levels [53].

### bMEC-related genes in SCC

No further indications for the self-arming of bMEC or their call for the assistance by professional immune cells could be detected during the first IM period. During the second IM period, the chemokines *CCL20* and *CXCL3* stuck out with a persistent down-regulation in the treated cows towards the Ctr. These chemokines released by bMEC would be aimed at the activation of lymphocytes and phagocytes, respectively [58–60]. However, frequent antigen challenging next to the mammary gland, as was implemented by the repeated PC inoculations in the area of the supramammary LNs, could have caused the bMEC’ habituation on these stimuli. Since many of the investigated phagocyte-related genes, like the complement receptors and the analyzed phagocyte cytokines, were also reduced transcribed in HR and LR, the innate IS should presumably be calmed overall during the second IM period.

### Gene expressions of phagocytes and lymphocytes in SCC

Following treatment with *MucoCD*-*I* batch A, the phagocyte-related genes were expressed rarely different between the groups, although the prevalent up-regulation of phagocyte receptors was conspicuous in HR and LR until the end of the first IM period. This could indicate a regeneration of phagocyte numbers, mainly of PMNs, in the mammary gland tissue after their previous consumption due to the removal of *C. difficile* antigens inoculated in TW 3, as is especially visible for the LR (Additional file 5: Table S5). This assumption could not be depicted by a significant down-regulation of these genes after this vaccination date but by corresponding data of the DCC (Additional file 5: Table S5) in TW 13. Although *IL1β*, *IL6* and *TLR2* were differently expressed by HR and LR each once and on separate dates during the entire treatment period, none of the examined phagocyte-related genes in the SCC were found to explain the basically different responsiveness in the treated groups. For this, the reactions within the specific IS challenged by the administered *C. difficile* vaccines seemed to be rather decisive. At the beginning of the treatment, the treated cows responded differently to vaccine batch A, as determined for the lymphocyte surface determinants *CD4* and *CD38*. Both were clearly up-regulated in the SCC of the LR towards the HR. *CD4* positive T cells can become the predominant phenotype in milk following their increased recruitment in the mammary gland during inflammatory reactions. One of their central functions is the activation of B cells in response to recognized antigens [61]. Within human breast milk cells, *CD38* displays the phenotypic hallmark of activated B lymphocytes [51]. The presence of both lymphocyte populations might have encouraged a further differentiation of B cells to generate antibody secreting cells. However, the humoral response in terms of increased anti-*C. difficile* IgA production failed to appear, and accordingly, the plasma cell marker *CD126* was finally lower expressed by the LR than by the other groups. Additionally, *IL10*, known to promote the humoral response, was repeatedly down-regulated in the treated groups during the first IM period [62]. Presumably, in favor of the cellular immunity, the *CD8β* positive lymphocytes were activated, which had shown the tendency towards their up-regulation in LR SCC from TW 3, and this alteration was verified from TW 8. *CD8β* positive T cells are the main lymphocyte subpopulation in the mammary gland and its secretions. They are able to act in a cytotoxic as well as suppressive way. *CD8β* positive T cells of the suppressive type are frequently responsible for the hypo-responsiveness of the IS of the mammary gland confronted with intramammary pathogens, like *Staphylococcus aureus* [48]. The TW 8 was also marked by high SCC levels (Fig. 5) of LR, which are generally a crucial clinical symptom of intramammary infections. The increase of SCC resulted possibly in the course of a counter-attack by *CD8β* T cells. Conflictingly, one TW later, *CD8β* of the HR was also increased, expressed towards the Ctr. The persistently inverse *IFNγ* regulations by the treated groups within the first treatment period could affirm the theory of pronounced cell-mediated immune responses developed by the LR (Fig. 4). *IFNγ* is a crucial intermediator between the innate and the adaptive IS by promoting the anti-microbicidal activity of phagocytes and the up-regulations of MHC-I and MHC-II molecules each for antigen presentation [62]. In contrast to its expression in PBL by the treated cows, *IFNγ* was sustainably over-expressed by the LR compared to its reduction in the HR. One more time, this *IFNγ* expression pattern of the treated groups emerged also during the second treatment period following the triple vaccination. However, further different alterations of lymphocyte-related genes between the treated groups could not be measured in the course of the treatment with *MucoCD*-*I* batch B.

### Total and differential cell counts in milk

The development of the total SCCs in milk seemed not to be impaired by the treatment and remained constantly beneath the upper threshold except for the outlier measured in LR milk in TW 8 (Fig. 5). By SC vaccination unaffected concentrations of milk SCCs were also found in a study evaluating the efficacy of a *Staphylococcus aureus* bacterin against mastitis in a lactating cow model [63]. Therefore, it can be assumed that vaccinations beyond the mammary gland generally do not interfere with the cellular homeostasis of the lacteal secretions, provided that the blood-udder barrier is intact [64]. The ratios of phagocytes to lymphocytes in milk did not vary between groups, as proven for the first IM period (Fig. 5). However, in terms of the normal level of this cell ratio in milk > 1, it should be noted that this was not applicable either for any group up to TW 6 and the LR group showed consistently cell ratios < 1 on any date of the first IM period. The examination of the differential SCCs revealed strikingly low proportions of macrophages, being usually below 10%, whereas this cell population out of the milk of healthy udders is known as the dominating share of the DCC (Additional file 5: Table S5) [31, 65]. Technical factors could have influenced the DCC in milk. Exemplarily, polyethylene plastic bottles were used for milk sampling, which might have minimized the macrophage population by adherence [65]. Furthermore, the microscopic evaluation of the different somatic cell populations could have understated the PMN fraction [66]. The outset of the second IM period was characterized by physiologically positive charged values for the ratio of phagocytes to lymphocytes in the milk of all treated cows (Fig. 5), but following the application of *MucoCD*-*I* batch B, this milk cell ratio diverged between HR and LR on numerous dates. While the HR could maintain the cell ratio within the normal range, the LR lost proportionally more lymphocytes than phagocytes in their lacteal secretions up to TW 23 (Fig. 5, Additional file 5: Table S5). Regarding the development of the milk lymphocyte concentrations in relation to their initial values, the delta values for the LR lymphocyte parts tended to increase while that of the HR showed an inverse drift subsequent to the PC injection of vaccine batch A (Fig. 3). Their contrary development led to clear differences between the treated groups in TWs 6 and 11. After treatment with vaccine batch B, considerably enhanced lymphocyte proportions were determined within the LR SCCs, which resulted in significant discrepancies towards the predominantly constant HR lymphocyte parts up to the end of the treatment. However, a unilateral attribution of the growth of the LR lymphocyte percentages to the vaccination program of the second IM period has to be doubted for two reasons. Firstly, the reference value used for the LR lymphocyte percentage in TW 16 was extraordinary low, which had a critical impact on the calculated delta values (Additional file 5: Table S5). Secondly, the extent of their increase seemed to be curious when taking into account that the likewise PC administered vaccine batch A, containing the imbalanced and poorer tolerated mixture of the *C. difficile* toxins and toxoids, triggered no significant altered lymphocyte concentrations in LR milk related to the baseline (TW 1). In general, the DCCs in the milk of all investigated groups indicated to be shaped by the physiological development of the milk cell populations in the course of lactation. As commonly specified for healthy cows with low SCC milk and as is particularly obvious in the Ctr group, the phagocyte portion grows, whereas the lymphocyte content declines as lactation progresses (Additional file 5: Table S5) [66].

## Conclusions

To sum up, the total immune cell counts in blood but not in milk of HR and LR were altered by the vaccination with *MucoCD*-*I* batch A. The variations of the DCCs in blood and milk between the treated groups were not unequivocally attributable to the IM against *C. difficile*. To evaluate the DCC, shortcomings of the selected examination method, such as light microscopy, could not be excluded. The gene expression analysis of PBL spawned sustainable differences between HR and LR. In particular, their expression patterns of the examined cytokines differed significantly during the first IM period. This outcome might have been crucial for the different response of the treated groups to the vaccinations against *C. difficile* because the intercellular mediators are important to orchestrate the immune cells for thwarting a pathogen attack. In doing so, *IFNγ* seemed to be a key player, as it stood out as a potent influencing factor of the local response in the mammary gland, too. The reason for the inversely expressed *IFNγ* in the SCC towards the PBL of the treated groups remained open. By confrontation with the *C. difficile* antigens, the LR seemed to be targeted at a stronger cellular immunity of the mammary gland in contrast to the HR. This will be proven by immunohistological examinations of different lymphocyte populations settled in the udder parenchyma. As analyzed in the SCC, the low anti-*C. difficile* IgA level in LR milk seemed not to be dependent on the limited transport capacity of the associated epithelial receptor *PIGR*. This was not found as differently regulated between the treated groups. Unlike the LR, more anti-*C. difficile* IgA secreting plasma cells had possibly been attracted for homing in the HR mammary gland due to the vaccination schedule. A subsequent immunohistological analysis will verify this assumption.

## Methods

### Animals and vaccination

The government of Upper Bavaria permitted the animal trial (AZ 55.2-1-54-2532.6-17-2012), as described below. During a 31-week treatment period, nine early lactating *Brown Swiss* cows were repeatedly vaccinated against *C. difficile*. The *MucoCD* vaccines (IDT Biologika, Dessau-Roßlau, Germany) comprised the crucial virulence factors of *C. difficile,* formaldehyde-inactivated whole cells (strain VPI 10463) and the exotoxins, TcdA and TcdB, being partly available as toxoids. More detailed information about their composition is subject to the company secret. For stimulating the mucosal IM system, the *MucoCD*-*N* vaccine was used for the biweekly nasal inoculation. With purpose of systemic IM, the *MucoCD*-*I* vaccine was injected via PC, IC or SC routes. The PC application was close to both supramammary LN. Two batches of *MucoCD*-*I* were administered. In TW 3, a single PC injection of vaccine batch A caused undesirable side effects and was consequently discharged. After the receipt of vaccine batch B, the IM via injection was administered in TW 17. The PC and the SC or IC vaccinations were scheduled at 4 weekly intervals. The detailed description of the IM routines was released by Schmautz et al. [21]. The treated cows were divided into two groups due to their immune responsiveness. The anti-*C. difficile* specific IgA content in milk was the distinctive feature and, at the end of the treatment, its total average value in cow’s milk determined the group membership. Treated cows with more than 8.0 µg ml^−1^ anti-*C. difficile* specific IgA in milk belonged to the high responder (HR) group (n = 5), those with lower anti-*C. difficile* specific IgA in milk formed the low responder (LR) group (n = 4). The control group (Ctr) consisted of five early lactating *Brown Swiss* cows supplied by the research station Veitshof of the Technical University of Munich (TUM, Freising, Germany).

### Milk sampling and preparation

Milk samples were taken once a week up to TW 8. Afterwards, they were collected biweekly. During the morning milking process, 500 ml of milk per cow were branched off with a TRU-Test milk meter (Lemmer-Fullwood, Lohmar, Germany), and then transferred to a wide-mouth polyethylene plastic bottle. A tenth of the milk volume taken was used for the SCC determination using optical fluorescent technique (*Fossomatic*-*FC* device, FOSS, Hamburg, Germany) by the contracted Milchprüfring Bayern association of Wolnzach, Germany. Out of 250 ml refrigerated raw milk the included somatic cells were extracted according to the following procedure: after centrifugation (1800×*g*, 4 °C, 15 min) the milk sample was defatted, the supernatant discarded, and the gained cell pellet re-suspended in 25 ml phosphate buffered saline (PBS, pH 7.4). Once again, the cell suspension was centrifuged with lower rotation speed (400×*g*, 4 °C, 10 min). This washing was repeated one more time. Afterwards, for the filtration step, only 10 ml of PBS were admitted to the cell pellet and then the cell suspension was percolated through a cell strainer (mesh size: 100 µm pores, Corning Inc., Corning, NY, USA). The filtrate was centrifuged (400×*g*, 4 °C, 10 min) and, after removal of the supernatant, the cells were aspirated with 250 µl of PBS. This cell extract was stored on ice until the preparation of the milk smears and the RNA extraction. For the IgA analysis, 20 ml of the raw milk sample were defatted (4000×*g*, 4 °C, 15 min), apportioned to 2-ml tubes and refrigerated at − 20 °C until further processing.

### Blood sampling and preparation

In the first 8 treatment weeks, 9 ml of blood of the cow jugular vein were sampled weekly and, in the remaining treatment period, their collection was performed in a regular 2-week cycles. EDTA pre-coated vacuettes (Greiner Bio-One GmbH, Frickenhausen, Germany) were used and put immediately on ice after the sampling. The blood stabilization was belatedly upgraded by addition of 100 µl of 0.3 M EDTA [33.5 g Titriplex III (Merck KgA, Darmstadt, Germany) dissolved in 300 ml of double distilled water and supplemented with 1% acetylsalicylic acid (Merck)]. PBL were extracted out of the blood as follows: after transferring 7 ml of EDTA blood into a 50-ml tube, 14 ml of lysis buffer [8.3 g NH_4_Cl (Carl Roth GmbH & CoKG, Karlsruhe, Germany), 0.37 g of Titriplex III (Merck) and 1.0 g of KCl (J.T. Baker Chemical Co. Phillipsburg, NJ, USA) dissolved in 1 l of double distilled water, pH 7.4] were added, intended for the lysis of the erythrocytes. The tube was carefully inverted three times and placed on ice until the dark red coloring of the suspension was visible. The PBL were gained after the centrifugation (400×*g*, 4 °C, 10 min) and the decantation of the supernatant. The following washing routine of the cells was done twice with 14 ml of lysis buffer in each case. Finally, the cell pellet was re-suspended in 2 ml of PBS, then the cell suspension was equally splitted into two 1.5 ml tubes and both tubes were placed on ice, one tube retained for RNA extraction and the other one for total PBL counting by using the TC10 automated cell counter (Bio-Rad Laboratories GmbH, Munich, Germany).

### *Clostridium difficile* specific IgA in milk

The particular procedure of the used sandwich ELISA determining the *C. difficile* specific IgA in cow milk can be looked up in the previous publication by Schmautz et al. [21]. In brief, a dilution series of the standard (1.76 mg ml^−1^ of *C. difficile* specific IgA, MucoVax b. v., Leiden, Netherlands) and a thinning of the milk samples at a ratio of one to ten were prepared with PBS. In duplicates, 100 µl standard dilutions or milk samples per well were applied to 96-well plates (Nunc MaxiSorp™, Sigma-Aldrich Chemie GmbH, Munich, Germany) pre-coated with *C. difficile* cells (2.0 × 10^8^ cells ml^−1^, IDT Biologika, Dessau, Germany). The incubation lasted for 1.5 h at 37 °C and was followed by multiple rinses with wash buffer. HRP conjugated sheep anti-bovine IgA (diluted 1:70,000, Bethyl Laboratories, Inc.; Montgomery, TX 77356, USA) was used as secondary antibody (100 µl well^−1^) and allowed to take effect under shelter for 1.5 h at 37 °C. Multiple washings were done before the TMB substrate mix was added (150 µl well^−1^) visualizing the antibody reactivity. After 40 min at RT, the chromogenic reaction was finished by adding the stop solution (50 µl well^−1^). The absorbance of the colored products were measured at 450 nm with a microplate reader (Sunrise™, Tecan Group Ltd., Männedorf, Switzerland). The concentration of *C. difficile* specific IgA in every milk sample was quantified relative to the standard curve by the software Magellan™ V6.6 (Tecan) belonging to the photometer.

### Differential cell counting (DCC) of milk and blood smears

For the milk smears, 20 µl of the prepared somatic cell-extract out of milk were pipetted on a standard microscope slide (76 × 26 mm, partly with frosted edge) in a meandering shape. For the blood smears, one droplet (3 µl) of the EDTA blood sample was spread out on a microscope slide with the narrow edge of a second one. Both, the milk and blood smears, were air dried and then processed with the Haema-Quick-Stain Kit (Diff-Quick; Labor + Technik, Eberhard Lehmann GmbH, Berlin, Germany) according to the manufacturer’s recommendations, producing staining results equivalent to the Pappenheim method. On completely dried smears, coverglasses (60 × 24 mm) were stuck with two droplets of the mounting media Eukitt^®^ (Sigma-Aldrich Chemie GmbH, Munich, Germany) per slide conserving the smear up to the microscopic differentiation. The evaluation was performed by light microscopy (Axioskop 2 plus, Carl Zeiss Microscopy GmbH, Göttingen, Germany). Using a “battlement track” method, between 200 and 300 cells were differentiated at 400-fold magnification for the blood smears, or at 1000-fold magnification and with oil immersion for the milk smears. The percentages of the various cell types in both body fluids were calculated in relation to the particular total cell counts of the samples.

### Total RNA extraction out of SC and PBL and transcription to cDNA

The miRNeasy Mini Kit (Qiagen GmbH, Hilden, Germany) was used to reap the RNA out of milk cells and PBL. For this purpose, 230 µl of somatic cell-extract or 1 ml of PBL suspension, were centrifuged for 5 min at 400×*g* and RT, respectively. After disposal of the supernatants, every gained cell pellet was lysed with 700 µl of Qiazol lysis reagent (Qiagen). Following cell disruption and homogenization for 5 min at RT, the samples could be stored interim at − 80 °C. The frozen homogenates were thawed at RT, continuing the RNA extraction in accordance with the manufacturer’s instructions. The total RNA was collected in 25 µl of RNase-free water departing from the manufacturer’s recommendation. The yielded RNA samples were immediately placed on ice. The total RNA concentration and its purity were determined at 260 nm with the NanoDrop ND-1000 (Peqlab, Erlangen, Germany). By using the 2100 Electrophoresis Bioanalyzer Instrument and the RNA 6000 Nano Assay Kit (Agilent Technologies, Waldbronn, Germany), the RNA integrity was examined. Until further processing, the RNA samples were stored at − 80 °C. For the reverse transcription of RNA to complementary DNA (cDNA), 300 ng or 1000 ng of RNA from each sample containing milk cells or PBL were used, respectively. The master mix included 5× buffer, 50 μM of random hexamers (Invitrogen Life Technologies, Darmstadt, Germany), 40 mM of dNTP Mix and 200 units of M-MLV reverse transcriptase (RNase H Minus, Point Mutant; Promega, Mannheim, Germany). At the ratio of one to three, the template and the master mix were mixed in 96-well plates (4titude^®^ Ltd., Berlin, Germany) to achieve a total volume of 60 µl of cDNA per sample. The thermal cycling of the reverse transcription was conducted by using the TPersonal Thermocycler (Biometra GmbH, Göttingen, Deutschland). After preheating the cover of the Thermocycler up to 104 °C, the single heating steps of the thermal cycling program were annealing for 10 min at 21 °C, transcription for 50 min at 48 °C and degradation of the reverse transcriptase for 2 min at 90 °C. The entrained positive controls were RNA extracts out of bovine mammary gland tissue and bovine spleen tissue to check the effective conducting of the following RT-qPCR. Excluding of non-inherent DNase in the RNA samples, a pooled sample with RNA out of all samples for examination was carried along without adding the reverse transcriptase. RNase-free water was used as a non-template control assuring a reverse transcription of the RNA without contaminations. The sealed cDNA containing multi-well plates were stored at − 20 °C.

### Primer design

For the detection of transcripts of 35 target genes and seven reference genes (*ACTG1, UB3, SUZ12, GAPDH, H3F3A, YWHAZ,* and *KRT8*), the corresponding bovine primer pairs were designed utilizing the Primer-BLAST of the National Center for Biotechnology Information (NCBI, National Library of Medicine, Bethesda, MD, USA, https://www.ncbi.nlm.nih.gov/tools/primer-blast/, accessed 07 Mar 2017.). The applied primers and the belonging details are compiled in the Additional file 1: Table S1. In each case, the primers functionality and optimal annealing temperature of 60 °C were tested with cDNA of the named positive control tissues using the RT-qPCR. The following primer deviated from the intended annealing temperature: *CCR6* (58 °C); *IL10*, *CD126*, *CD163* and *PIGR* (62 °C); *SUZ12* (64 °C). The amplification efficiency of each primer was tested pursuant to the Minimum Information for Publication of Quantitative Real-Time PCR Experiments (MIQE) guidelines. Only primers showing > 85% amplification efficiency in the RT-qPCR were used for the following gene expression measurements [67]. Furthermore, the primer specificity verified with gel electrophoresis of the PCR products was part of the quality assessment prior to usage of the primers.

### Gene expression measurements

The RT-qPCR was performed with the Rotor-Gene Q cycler (Rotor-Disc 72; Qiagen GmbH, Hilden, Germany). The reaction components, 1 µl of cDNA template, 5 µl of SsoFast EvaGreen Supermix (Bio-Rad Laboratories GmbH, Munich, Germany), 400 nM forward and reverse primers (metabion international AG, Planegg, Germany) filled up with DNase-free water to a final volume of 10 µl, were poured into the cycler accessory strip tubes (0.1 ml, Qiagen GmbH, Hilden, Germany). The cycler was operated by the related Rotor-Gene Q software version 6.0.38 (Corbett Research 2004, acquired by Qiagen GmbH, Hilden, Germany). The temperature profile of the RT-qPCR comprised first of all 30 s at 98 °C, followed by 40 cycles of two steps with 5 s at 95 °C and 20 s at the primer specific annealing temperature, and finally, generating the melting curve up to 95 °C in one-degree increments. The PCR products were preserved frigidly at 8 °C.

### RT-qPCR data processing

Based on the assay information outputted by the Rotor-Gene Q software (Qiagen GmbH, Hilden, Germany), the PCR performance was evaluated considering the MIQE Guidelines [67]. The PCR raw data for analysis are the generated Cq, which define the cycle number as take-off point for the detection of the specific fluorescence signal. The relative quantification of the PCR signals required a set of reference genes for normalization. The GenEx ‘Normfinder’ tool (GenEx software version 6.1; MultiD Analyses AB, Gothenburg, Sweden) extracted seven possible reference genes out of the raw data of all examined genes. For the normalization, six of the suggested seven reference genes were regularly used. The seventh one, KRT8, was respected only for the bMEC-related genes to be found in milk samples. After the interplate calibration based on the entrained and sample source relevant positive controls, the difference between the mean Cq value of all considered reference genes and the target gene-specific Cq was calculated per sample (ΔCq). The relative changes in gene expression to designated dates was calculated with the 2^−ΔΔCq^ method [68].

### Statistics

The data of each individual animal were assigned to the related group time-dependently. Variations within or between groups occurring during the treatment were figured using the MIXED procedure model in SAS/STAT^®^ 9.22 (2010 SAS Institute Inc., Cary, NC, USA). After checking the convergence criteria by the log-likelihood calculation, the estimated least square means (LSM) plus standard errors (SD) were released by the applied statistic software. For all statistics, the significance level at *p* < 0.05 was decisive.

## Additional files

**Additional file 1: Table S1.** Primer used for the RT-qPCR examinations. The primer construction based on the nucleotide sequences provided by the gene database of the National Center for Biotechnology Information (NCBI, https://www.ncbi.nlm.nih.gov/nuccore/, accessed 07 Mar 2017). The associated NCBI reference sequence numbers are listed in the Additional file 1: Table S1.

**Additional file 2: Table S2.** ΔΔCq changes in gene expression of PBL relative to TW 0 or rather TW 16.

**Additional file 3: Table S3.** ΔΔCq changes in gene expression of SC relative to TW 1 or rather TW 15.

**Additional file 4: Table S4.** Total PBL counts and the relative distribution of the different leukocyte populations.

**Additional file 5: Table S5.** Total SC counts and the relative distribution of the different leukocyte populations in milk.

## Abbreviations

APCs: antigen presenting cells
bMEC: bovine mammary epithelial cells
CDI: *C. difficile* infection
CDAD: *C. difficile* associated diarrhea
Cq: quantitation cycle
Ctr: control
DCC: differential cell counting
HR: high responder
IC: intracutaneous
iFA: incomplete Freund’s adjuvant
IM: immunization
IS: immune system
LN: lymph node
LR: low responder
MHC: major histocompatibility complex
PAMPs: pathogen-associated molecular patterns
PBL: peripheral blood leukocytes
PC: percutaneous
PMN: polymorph nucleated neutrophil
PRR: pattern recognition receptor
RT: room temperature
RT-qPCR: real time quantitative PCR
SC: subcutaneous
SCC: somatic cell count
TcdA: *C. difficile* toxin A
TcdB: *C. difficile* toxin B
TCR: T cell antigen receptor
T_C_ cell: cytotoxic T cell
T_H_ cell: T helper cell
TW: treatment week
