# Supplementary material for: Immune cell counts and signaling in body fluids of cows vaccinated against Clostridium difficile
Source: J Biol Res (Thessalon). 2018 Dec 10;25:20. doi: 10.1186/s40709-018-0092-4 (PMC6288880; doi:10.1186/s40709-018-0092-4)
Supplement: Supplementary file 1 — Additional file 1: Table S1. Primer used for the RT-qPCR examinations. The primer construction based on the nucleotide sequences provided by the gene database of the National Center for Biotechnology Information (NCBI, https://www.ncbi.nlm.nih.gov/nuccore/, accessed 07 Mar 2017). The associated NCBI reference sequence numbers are listed in the Additional file 1: Table S1. [file 40709_2018_92_MOESM1_ESM.pdf]

Additional file 1

Immune cell counts and signaling in body fluids of cows vaccinated against *Clostridium difficile*.

Christiane Schmautz[1], Nadine Müller[1], Marlene Auer[1], Ines Ballweg[1], Michael W. Pfaffl[1] and Heike Kliem [1]*

[1] *Chair of Animal Physiology and Immunology, Technical University of Munich (TUM), Weihenstephaner Berg 3, 85354 Freising, Germany*

* Corresponding author. E-mail: heike.kliem@wzw.tum.de

Table S1. Primer used for the RT-qPCR examinations.

**Gene name (alternative names) (*acronym*)**	**NCBI[1] reference sequence number**	**Primer sequences (5' → 3'), forward[2] and reverse[3]**	**Length [bp][4]**
Reference genes			
Actin gamma 1 (*ACTG1*)	NM_001033618	AACTCCATCATGAAGTGTGAC	234
		GATCCACATCTGCTGGAAGG	
Polyubiquitin (*UB3*)	Z18245	AGATCCAGGATAAGGGAAGGCAT	198
		GCTCCACCTCCAGGGTGAT	
SUZ12 polycomb repressive complex 2 subunit homolog (*SUZ12*)	NM_001205587.2	AGCCATGCAGGAAATGGAAG	183
		GCAAGAGGTTTGGCTATAGG	
Glyceraldehyde-3-	NM_001034034.2	GTCTTCACTACCATGGAGAAGG	197

phosphate dehydrogenase (*GAPDH*)		TCATGGATGACCTTGGCCAG	
H3 histone, family 3A (*H3F3A*)	NM_001014389.2	ACTTGCTACAAAAGCCGCTC	232
		ACTTGCCTCCTGCAAAGCAC	
Tyrosine 3-monooxygenase/ tryptophan 5-monooxygenase-activation protein zeta (*YWHAZ*)	NM_174814.2	CAGGCTGAGCGATATGATGA	141
		GACCCTCCAAGATGACCTAC	
Cytokeratin 8 (*KRT8*)	NM_001033610.1	TGGTGGAGGACTTCAAGACC	215
		CGTGTCAGAAATCTGAGACTGC	
Cell surface receptors			
T-cell surface glycoprotein CD3 delta chain (*CD3δ*)	NM_001034033.2	CTGCCCTTCTCTCCCAAGTG	301
		ACAGTGGCAATGATGTCGGT	
T-cell surface glycoprotein CD4 (*CD4*)	NM_001103225.1	TCCCAATCCTGGGTCACCT	159
		ATCCAGGGTCAGGGTTCCA	
T-cell surface glycoprotein CD8 beta chain (*CD8β*)	NM_001105344.2	TTCTGGTGTCCTTGGGTGTG	189
		ATTTCCACGGCTGAGTGTGT	
B cell surface marker CD19 (*CD19*)	NM_001245998.1	CTGTGCTCCACCTAACAGCA	110
		TGACCAGGGAGGTATGGGAG	
B- and T-cells related ADP-ribosyl cyclase 1	NM_175798.3	TCGTTGGAGAACGCCTTTGA	137
		GAGGAGCCAGAGCATGAGTC	

(*CD38*)			
Activated B- and plasma cells associated interleukin 6 receptor subunit alpha (*CD126*)	NM_001110785.1	GGGATCAGATGACAGGCTCG	258
		GTAACACGGCCTTGGTGGTA	
Monocytes and macrophages assigned scavenger receptor cysteine-rich type 1 protein M130 (*CD163*)	NM_001163413.1	CGAGTCCCATCTTTCACTCTG	285
		AGTGAGAGTTGCAGAGAGGTCC	
Toll like receptor 2 (*TLR2*)	NM_174197.2	CATTCCTGGCAAGTGGATTATC	201
		GGAATGGCCTTCTTGTCAATGG	
IgG Fc receptor (*FcRN*)	AF141017.1	GAGCTGGCTCCTTGGATCTC	194
		ATACCAGGATTCCCGGAGGT	
Polymeric immunoglobulin receptor (*PIGR*)	NM_174143.1	TGCGACCAGAACAGCCAG	278
		CCAGCAGCGTCCTTCACA	
Cytokines			
Interferon gamma (*IFNγ*)	NM_174086.1	TGGCAGCTCTGAGAAACTGG	199
		CAGGCAGGAGGACCATTACG	
Interleukin 1 beta (*IL1β*)	NM_174093.1	CAGTGCCTACGCACATGTCT	209
		AGAGGAGGTGGAGAGCCTTC	
Interleukin 2 (T-cell growth factor) (*IL2*)	NM_180997.2	TCAAGCTCTACGGGGAACAC	146
		GTAGCGTTAACCTTGGGCAC	
Interleukin 6 (Interferon beta 2) (*IL6*)	NM_173923.2	TGGTGATGACTTCTGCTTTCC	109
		AGAGCTTCGGTTTTCTCTGG	

Interleukin 10 (Cytokine synthesis inhibitory factor) (*IL10*)	NM_174088.1	AGCTGTATCCACTTGCCAACC	119
		TGGGTCAACAGTAAGCTGTGC	
Interleukin 12 subunit beta (Natural killer cell stimulatory factor 2, Cytotoxic lymphocyte maturation factor 2, p40) (*IL12β*)	NM_174356.1	GGTTTTCCCTGGTTTTGCTGG	175
		ACCTCACTGCTCTGGTCTGA	
Tumor necrosis factor alpha (Cachectin) (*TNFα*)	NM_173966.3	CCACGTTGTAGCCGACATC	108
		ACCACCAGCTGGTTGTCTTC	
Transforming growth factor beta 1 (*TGFβ1*)	NM_001166068.1	CCTGGACACCAACTACTGCT	185
		CCAGGACCTTGCTGTACTGT	
Chemokines and their receptors			
Chemokine (C-X-C motif) ligand 3 (Epithelial cell inflammatory protein) (*CXCL3*)	NM_001046513.2	TACAGAGCGTGAAGGTGACG	164
		CCCTCTAGGTCAGTTGGTGC	
Chemokine (C-X-C motif) ligand 5 (Chemokine (C-X-C motif) ligand 6, Granulocyte chemotactic protein 2) (*CXCL5*)	NM_174300.2	TTGTGAGAGAGCTGCGTTGT	112
		ACTTCCACCTTGGAGCACTG	

C-X-C motif chemokine receptor 2 (Interleukin 8 receptor beta) (*CXCR2*)	NM_001101285.1	CAACACTGACCTGCCCTCTA<br>CCAGGTTCAGCAGGTAGACA	197
C-X-C motif chemokine ligand 8 (Interleukin-8, Neutrophil activating peptide 1) (*CXCL8*)	NM_173925.2	AAGAATGAGTACAGAACTTCGATGC<br>GTTTAGGCAGACCTCGTTTCC	160
C-C motif chemokine ligand 5 (T-cell-specific protein RANTES) (*CCL5*)	NM_175827.2	TCCATGGCAGCAGTTGTCTT<br>TTCAGGTTCAAGGCGTCCTC	129
C-C motif chemokine ligand 20 (Macrophage inflammatory protein 3 alpha, MIP-3-alpha) (*CCL20*)	NM_174263.2	CTTGTGGGCTTCACACAGC<br>GTTTCACCCACTTCTTCTTTGG	115
C-C motif chemokine receptor 6 (*CCR6*)	NM_001194961.1	TCATGAAGGACCTGTGGTGC<br>TGAAGGAAGACGGGTTGTCG	132
C-C motif chemokine ligand 28 (*CCL28*)	NM_001101163.1	TGACGGGGATTGTGACTTGG<br>CCGATGTGCCCCTTTACTGT	184
Complement proteins and their receptors			
Complement C1q A chain (*C1QA*)	NM_001014945.2	CGTTGGACCGAATTCTGTCTC<br>TGCTGTTGAAGTCACAGAAGCC	224
Complement C3 (*C3*)	NM_001040469.2	AAGTTCATCACCCACATCAAG<br>CACTGTTTCTGGTTCTCCTC	191
Complement C3a	NM_001083752.1	CCCTCCATCATCATCCTCAAC	167

receptor 1 (C3a anaphylatoxin chemotactic receptor 1) (*C3AR1*)		CACATTACCAAAGCCACCACC	
Complement C5a receptor 1 (C5a anaphylatoxin chemotactic receptor 1) (*C5AR1*)	NM_001007810.3	ATACCGTCCTTTGTGTTCCG<br>ATTGTAAGCGTGACCAGCG	158
Antimicrobial peptides			
Lactotransferrin (Lactoferrin) (*LF*)	NM_180998.2	CGAAGTGTGGATGGCAAGGAA<br>TTCAAGGTGGTCAAGTAGCGG	215
Lactoperoxidase (*LPO*)	NM_173933.2	TGGCTGTCAACCAAGAAGC<br>TGAGGCTCGAAAATCTCCC	134
Lysozyme 1 (Lysozyme C) (*LYZ1*)	NM_001077829.1	AAGAAACTTGGATTGGATGGC<br>ACTGCTTTTGGGGTTTTGC	185
Tracheal antimicrobial peptide (*TAP*)	NM_174776.1	AGGAGTAGGAAATCCTGTAAGCTGTGT<br>AGCATTTTACTGCCCGCCCGA	113

[1] NCBI = National Center for Biotechnology Information
(National Library of Medicine, Bethesda, MD, USA);
source of the used gene database (https://www.ncbi.nlm.nih.gov/nuccore/).

[2] Forward primer sequence are listed in the upper line.

[3] Reverse primer sequence are listed in the lower line.

[4] bp = base pairs
